# The Extraintestinal Pathogenic *Escherichia coli* Factor RqlI Constrains the Genotoxic Effects of the RecQ-Like Helicase RqlH

**DOI:** 10.1371/journal.ppat.1005317

**Published:** 2015-12-04

**Authors:** Colin W. Russell, Matthew A. Mulvey

**Affiliations:** University of Utah School of Medicine, Department of Pathology, Division of Microbiology and Immunology, Salt Lake City, Utah, United States of America; Osaka University, JAPAN

## Abstract

Extraintestinal pathogenic *Escherichia coli* colonize the human gut and can spread to other body sites to induce diseases such as urinary tract infections, sepsis, and meningitis. A complete understanding of the infection process is hindered by both the inherent genetic diversity of *E*. *coli* and the large number of unstudied genes. Here, we focus on the uncharacterized gene *rqlI*, which our lab recently uncovered in a Tn-seq screen for bacterial genes required within a zebrafish model of infection. We demonstrate that the Δ*rqlI* mutant experiences a growth defect and increased DNA stress in low oxygen conditions. In a genetic screen for suppressor mutations in the Δ*rql* strain, we found that the shortcomings of the Δ*rql* mutant are attributable to the activity of RqlH, which is known in other bacteria to be a helicase of the RecQ family that contains a phosphoribosyltransferase (PRTase) domain. Disruption of *rqlH* rescues the Δ*rqlI* strain in both *in vivo* and *in vitro* assays, while the expression of RqlH alone activates the SOS response coincident with bacterial filamentation, heightened sensitivity to DNA damage, and an increased mutation rate. The analysis of truncation mutants indicates that, in the absence of RqlI, RqlH toxicity is due to its PRTase domain. Complementary studies demonstrate that the toxicity of RqlH is modulated in a context-dependent fashion by overlapping domains within RqlI. This regulation is seemingly direct, given that the two proteins physically interact and form an operon. Interestingly, RqlH and RqlI orthologs are encoded by a diverse group of bacteria, but in many of these microbes, and especially in Gram-positive organisms, *rqlH* is found in the absence of *rqlI*. In total, this work shows that RqlH and RqlI can act in a strain-specific fashion akin to a toxin-antitoxin system in which toxicity is mediated by an atypical helicase-associated PRTase domain.

## Author Summary

Extraintestinal pathogenic *Escherichia coli* (ExPEC) cause the majority of urinary tract infections, and are also able to infect the bloodstream, meninges, and various other sites within the human host. These infections are becoming increasingly difficult to treat as ExPEC strains gain resistance to many of the antibiotics that are commonly used in the clinic. The development of improved treatment strategies requires a deeper understanding of the factors that promote ExPEC fitness and virulence within the host. In genetic screens, we identified a functionally uncharacterized protein, RqlI, which promotes ExPEC survival within diverse host environments. We find that RqlI binds to and works in tandem with RqlH, a protein that has been shown in other bacteria to unwind DNA. In the absence of RqlI, we found that RqlH can become toxic to ExPEC, causing DNA damage and slower growth. A specific part of RqlH that is predicted to manipulate the nucleotides that make up DNA is responsible for this toxicity. The ability of RqlH to inhibit bacterial growth when not held in check by RqlI suggests that the specific inactivation of RqlI could have therapeutic value in combating ExPEC and other pathogens that express these proteins.

## Introduction

Extraintestinal pathogenic *Escherichia coli* (ExPEC) comprise a group of *E*. *coli* strains that harmlessly reside within the gastrointestinal tract, but that are capable of infecting extraintestinal niches such as the urinary tract, bloodstream, and meninges [1,2]. Urinary tract infections (UTIs) alone constitute a major global economic burden due to the high incidence rate of UTIs. Indeed, more than half of all women will experience a UTI during their lifetime [3], with bladder infections being responsible for more than $2 billion in medical care costs in the United States annually [4]. Additionally, *E*. *coli* is the leading cause of bacteremia [5], and is especially problematic in high risk, immunocompromised populations such as the elderly [6,7] and cancer patients undergoing chemotherapy [8]. *E*. *coli* is also the leading cause of death in early-onset neonatal sepsis [9]. The burden imposed by ExPEC infection is expanding as the prevalence of antibiotic resistant strains increases [10], highlighting the need for a deeper understanding of ExPEC-mediated pathogenesis.

A major hurdle in deciphering the ExPEC life cycle is the genetic heterogeneity inherent in these strains. Each ExPEC isolate contains ~5,000 genes, of which about half comprise a core genome that is shared with most other *E*. *coli* strains [11]. The genes outside of the core genome are much less conserved and account for the bulk of the genomic heterogeneity observed among ExPEC isolates. This flexible genome is in large part a consequence of horizontal gene transfer among ExPEC strains and other bacteria. The ExPEC pan-genome, which includes all distinct genes found within ExPEC isolates, contains at least 14,877 genes [11]. Due to the dynamic nature of the pan-genome, this estimate is likely to increase as more isolates are sequenced [12]. Because of the genetic differences from one ExPEC isolate to another it is difficult to define a specific set of genes that are universally required for ExPEC fitness.

Complicating matters further is the fact that many ExPEC-associated genes are functionally undefined. One method for dealing with the large number of uncharacterized genes is to employ high-throughput analyses that can assay the role of many genes in a given condition simultaneously. One especially useful technique is Tn-seq, in which a pool of transposon mutants is placed under a selective pressure, and the quantity of individual mutant variants before and after the selection are enumerated by next-generation sequencing [13]. Despite their utility, these types of analyses often result in lists of genes that are required for fitness under a given condition without yielding clear insight into the actual functions of individual genes. For example, in a recent Tn-seq screen for factors that affect ExPEC fitness in a zebrafish infection model, many of the 981 genetic elements that were found to be important for host colonization had never been studied [14]. Annotations for 152 of the 981 loci (15.5%) that were identified as required for zebrafish colonization include the words “hypothetical” or “putative”. In addition, given the limitations of the current pipelines used to annotate genes, many more of the identified loci are likely misannotated [15]. Thus, understanding why a gene is important for fitness under a given condition can be complicated. This problem can be partially remedied by comparing overlapping datasets from high-throughput experiments carried out in various defined conditions [16,17]. This strategy can help uncover roles for previously undefined genes, but additional detailed analysis is still required to validate the high-throughput data and to unambiguously specify gene function.

In our recent Tn-seq screen using the ExPEC cystitis isolate F11, we identified the hypothetical gene *EcF11_3933* as an important mediator of pathogen fitness during both localized and systemic infections within the surrogate zebrafish host [14]. This gene, which we propose to rename RecQ-Like Helicase Interactor (*rqlI*) for reasons that will become clear, was also critical to the ability of F11 to colonize the mouse urinary tract and for pathogen survival in a mouse model of sepsis. Considering the importance of *rqlI* to pathogen survival in diverse hosts and host environments, we set out to define the specific function of RqlI. Here we report that RqlI is especially critical for pathogen growth under low oxygen conditions, as found within many host niches. In the absence of RqlI, the bacteria have higher mutation rates, increased activation of the SOS stress response system, and decreased growth. These effects are remedied by deletion of *rqlH*, a gene immediately upstream of *rqlI* that encodes RecQ-like Helicase (RqlH). Deletion of *rqlH* also rescues the *in vivo* survival defects observed with the Δ*rqlI* mutant in several mouse models. Genetic and biochemical analyses indicate that a function of RqlI within ExPEC is to limit the genotoxic effects of RqlH, which we mapped to an unusual phosphoribosyltransferase (PRTase) domain localized at the C-terminus of RqlH. Interestingly, we find that RqlH and RqlI homologs are encoded by a diverse collection of bacterial species, though the *rqlH* and *rqlI* genes are not always paired as in F11. These observations suggest that RqlI can have both RqlH-dependent and RqlH-independent functions, and that these two proteins have important roles in the life cycles of a wide range of bacteria.

## Results

### The Δ*rqlI* mutant exhibits a growth defect in microaerobic conditions

**Fig 1:**
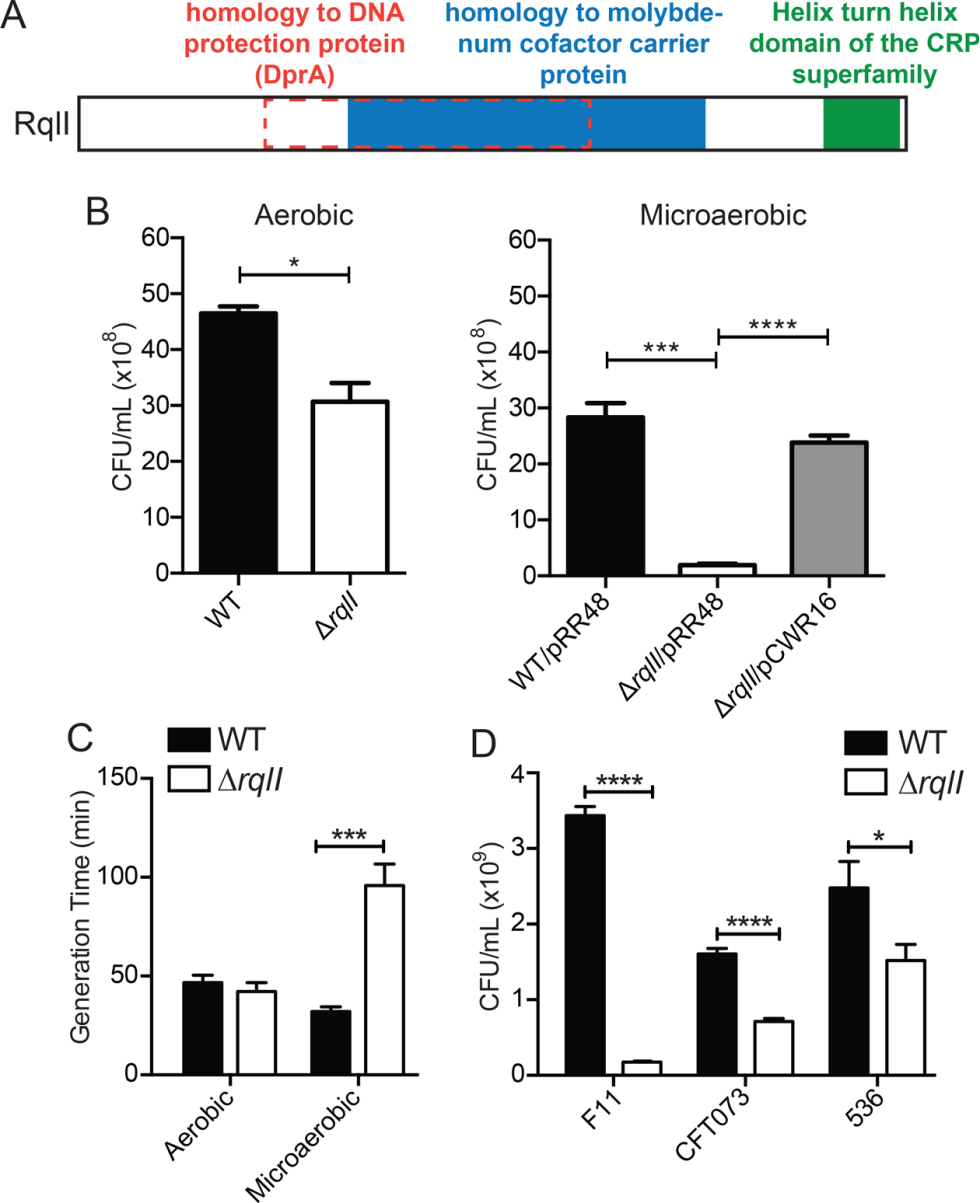
Growth of *rqlI* deletion mutants is severely impaired in low oxygen conditions. (A) A schematic of predicted domains and homology regions of RqlI. (B) WT F11 or F11Δ*rqlI* were grown either aerobically or microaerobically for 24 h in a modified M9 media, at which point the cultures were titered. Under microaerobic conditions, strains that carried either an IPTG-inducible *rqlI* expression vector (pCWR16) or the empty vector control (pRR48) were grown in the presence of IPTG. (C) WT F11 or F11Δ*rqlI* were grown either aerobically or microaerobically, and the cultures were titered at two different time points during exponential phase in order to calculate generation times. (D) The indicated WT and Δ*rqlI* mutant ExPEC isolates were grown microaerobically for 24 h and titered. Bars indicate mean values ± SEM from three or more independent experiments performed in triplicate. *, *P* ≤ 0.05; ***, *P* ≤ 0.001; ****, *P* ≤ 0.0001; as determined by Student’s *t* test.

An initial analysis of the RqlI protein revealed the presence of three putative domains (Fig 1A). First, RqlI is predicted to contain a molybdenum cofactor (MoCo) binding domain, which is often found in proteins that facilitate metabolism in low oxygen environments [18]. For example, MoCo facilitates redox reactions critical for using alternative terminal electron acceptors when oxygen is scarce. Second, overlapping the MoCo binding domain is a region with homology to the DNA-protecting protein DprA, which has been shown to be important for the uptake of exogenous DNA in naturally competent bacteria [19]. Third, at the C-terminus of RqlI is a helix-turn-helix motif predicted to bind DNA.

Given the involvement of MoCo in anaerobic respiration [18], and given the putative MoCo-binding domain contained within RqlI, we disrupted the *rqlI* gene in the reference ExPEC strain F11 and tested the ability of the mutant to multiply in varying oxygen levels. When grown for 24 hours in a modified M9 minimal media exposed to atmospheric oxygen (~20% O_2_), F11Δ*rqlI* exhibits a slight growth defect in comparison to the wild type (WT) strain (Fig 1B). However, in microaerobic culture conditions (6–12% O_2_) growth of the Δ*rqlI* mutant was markedly worse than WT (Fig 1B). This defect was rescued by complementation with the RqlI expression plasmid pCWR16. Furthermore, we found that the exponential phase generation time of the Δ*rqlI* mutant was very similar to WT cells under aerobic conditions, but was 3-fold greater than WT bacteria in a microaerobic environment (Fig 1C). Under microaerobic conditions, deletion of *rqlI* also limited the growth of two other reference ExPEC strains, CFT073 and 536 (Fig 1D).

### The SOS response is activated in the Δ*rqlI* mutant during microaerobic growth

**Fig 2:**
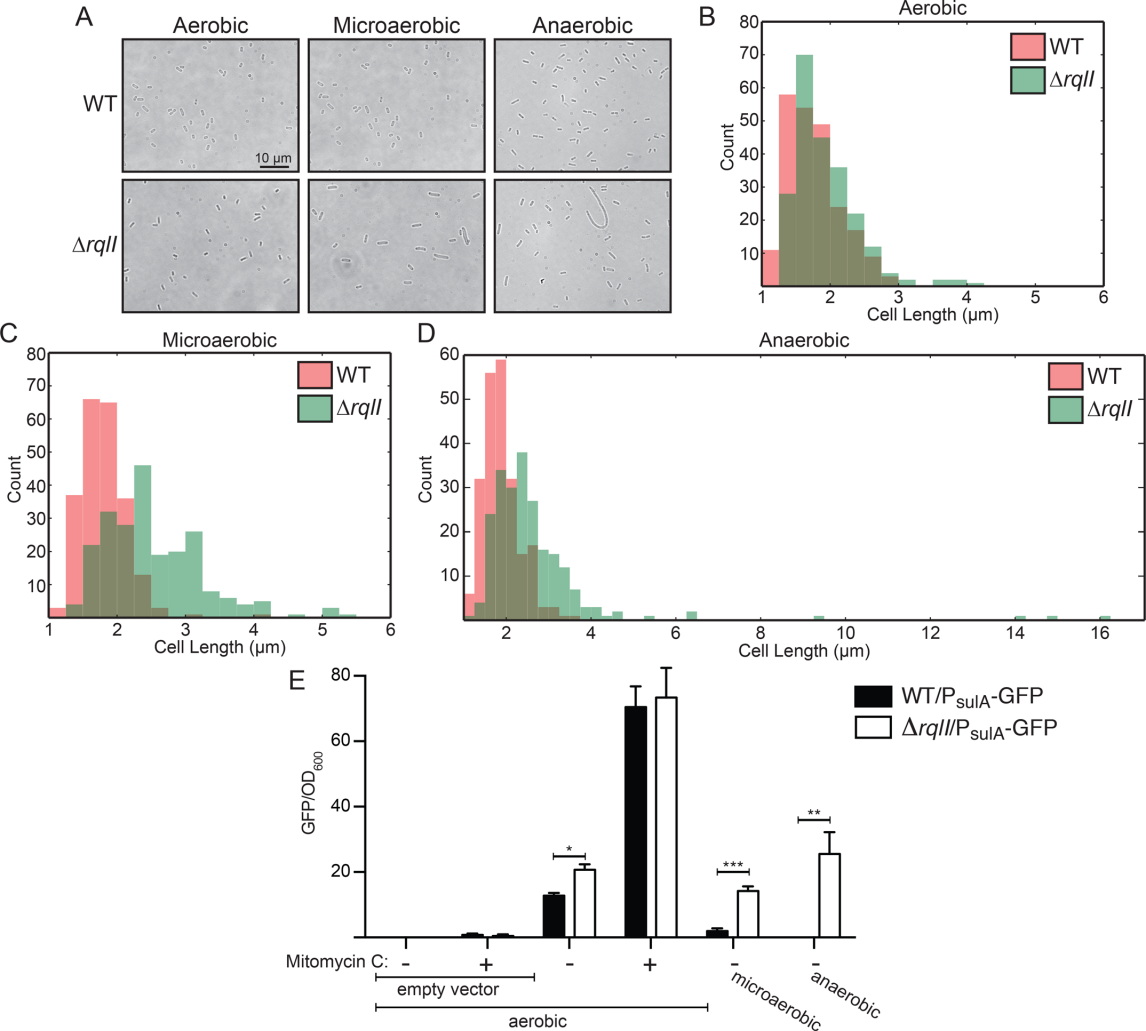
The *rqlI* mutant experiences increased genotoxic stress, especially under low oxygen conditions. (A-D) WT F11 or F11Δ*rqlI* were grown 24 h aerobically, microaerobically, or anaerobically, at which point bacteria were spread on a slide and imaged. (A) Representative images. (B-D) Histograms show the distribution of bacterial cell lengths after growth at varying oxygen levels. (E) WT F11 and F11Δ*rqlI* strains carrying either the P_*sulA*_-GFP reporter plasmid (pJLJ3) or an empty vector control (pJLJ1) were grown aerobically ± mitomycin C, microaerobically, or anaerobically, as indicated. Fluorescence measurements were normalized to culture densities. Bars indicate mean values ± SEM from three or more independent experiments performed in triplicate. *, *P* ≤ 0.05; **, *P* ≤ 0.01; ***, *P* ≤ 0.001; as determined by Student’s *t* test.

We examined the general morphology of F11Δ*rqlI* using light microscopy and noticed that the mutant cells were often longer (more filamentous) than WT bacteria (Fig 2A–2D). The elongated Δ*rqlI* mutant cells were especially abundant, and reached greater lengths, under microaerobic or anaerobic conditions (Fig 2B–2D). One reason for bacterial cell length to increase is the induction of the SOS response, which is initiated when DNA damage is sensed [20]. The SOS response involves the upregulation of *sulA* expression, which functions to temporarily inhibit cell division while DNA repair occurs. Using a P_*sulA*_ *-*GFP fluorescent reporter construct, we found that F11Δ*rqlI* expressed markedly higher levels of *sulA* than the WT strain in aerobic, microaerobic, and anaerobic conditions (Fig 2E). The differences in *sulA* expression levels between WT and the Δ*rqlI* mutant grew more pronounced with decreasing amounts of oxygen in the culture. In control samples, *sulA* expression levels in the WT and Δ*rqlI* strains were similarly increased upon treatment with the DNA-damaging agent mitomycin C. These fluorescent measurements were confirmed by western blots probed with GFP-specific antibody (S1 Fig). In total, these data demonstrate that in the absence of RqlI there is increased activation of the SOS response, coincident with an oxygen-sensitive decrease in bacterial replication.

### RqlH provokes the SOS response and inhibits bacterial growth in the absence of RqlI

**Fig 3:**
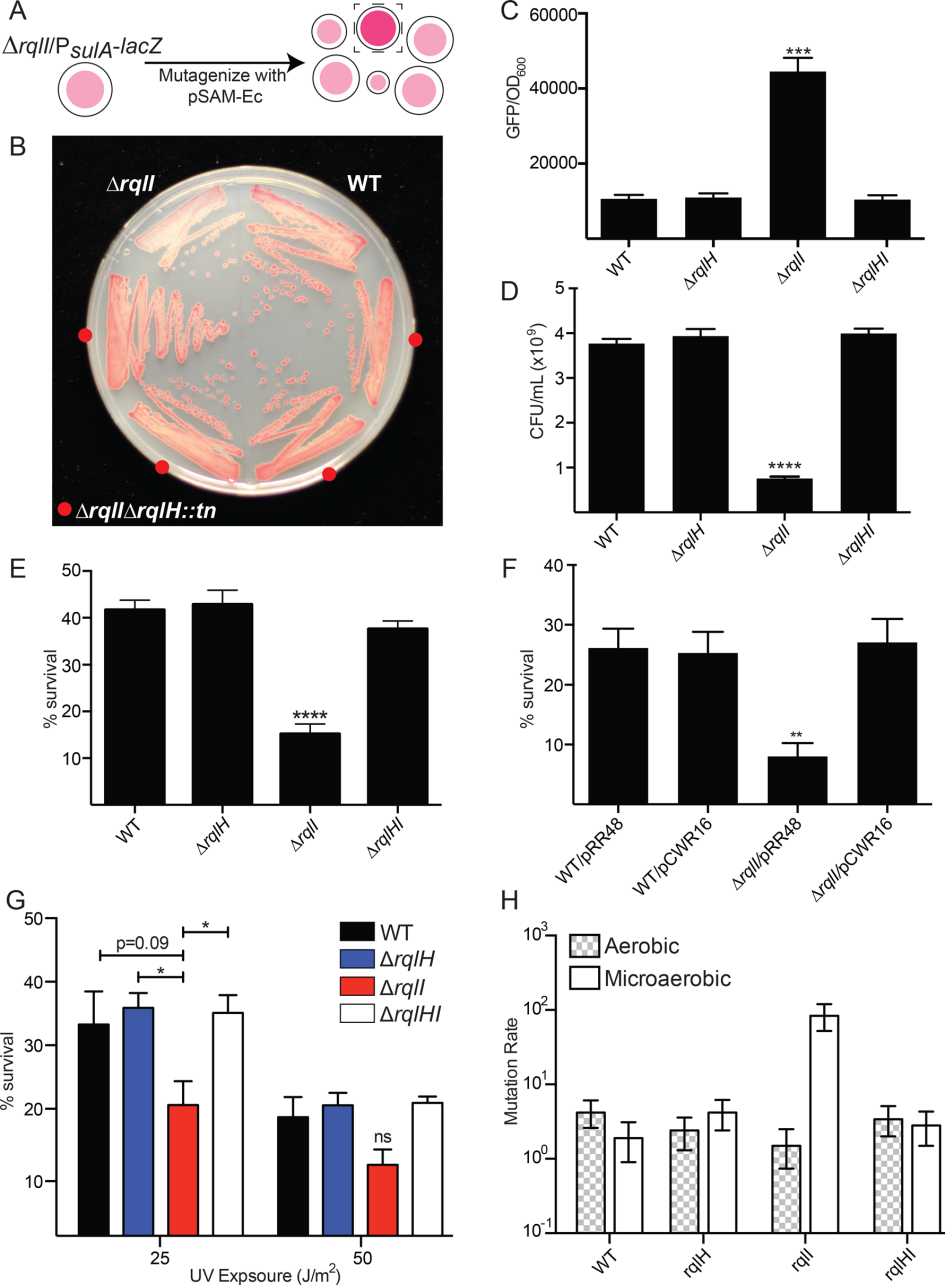
Mutation of *rqlH* rescues the *rqlI* mutant defect *in vitro*. (A) A schematic of the screen used to search for suppressor mutations of the Δ*rqlI* mutant. Bacteria carrying a P_*sulA*_ *-lacZ* reporter plasmid (pCWR2) were transposon-mutagenized and screened on tetrazolium lactose agar for suppressor mutants with decreased *sulA* promoter activity, (distinguishable by darker colony color). (B) A tetrazolium lactose plate demonstrating the colony colors for WT, F11Δ*rqlI*, and four independent F11Δ*rqlI*Δ*rqlH*::*tn* transposon mutants that were isolated from the screen. (C) Bacteria carrying pJLJ3 (P_*sulA*_-GFP) were grown microaerobically and GFP levels and culture densities were measured to quantify *sulA* expression. (D) Microaerobic growth of WT, Δ*rqlH*, Δ*rqlI*, and Δ*rqlHI* strains, as measured by dilution plating of 24 h cultures. (E) Bacteria were grown aerobically for 4 h, at which point mitomycin C was added to a final concentration of 0.25 μg/mL for 1 h. The cultures were titered immediately before and 1 h after addition of mitomycin C, and survival was calculated. (F) WT and Δ*rqlI* cells carrying either an empty vector control (pRR48), or the RqlI expression plasmid pCWR16 were grown and tested for mitomycin C survival as in (E). (G) Bacteria were grown aerobically for 4 h, then diluted and spread onto plates to get ~100 colonies per plate. The plates were then exposed to various levels of UV radiation and incubated overnight, at which time percent survival was calculated. In C-G, bars indicate mean values ± SEM from three or more independent experiments performed in triplicate. *, *P* ≤ 0.05; ***, *P* ≤ 0.001; ****, *P* ≤ 0.0001 in comparison with WT; as determined by Student’s *t* tests. (H) Bacteria were grown either for 4 h aerobically or 24 h microaerobically. Aliquots from each culture were used to titer viable bacteria, and the remaining cultures were plated onto agar containing nalidixic acid. The numbers of nalidixic acid resistant mutants recovered were used as input for FALCOR to calculate mutation rates (number of mutations per 10^10^ cells per generation). Bars indicate mean values ± 95% confidence intervals.

**Fig 4:**
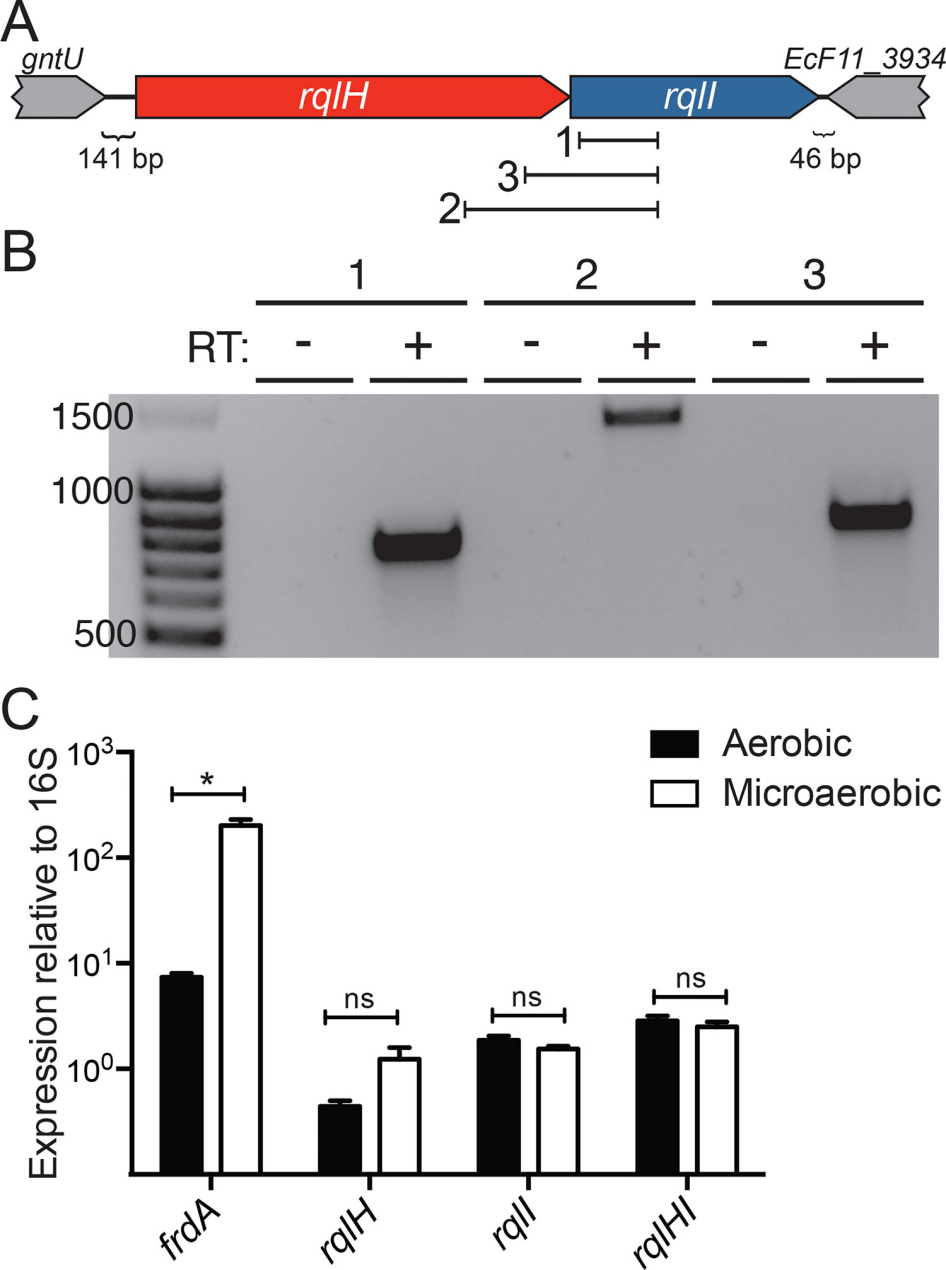
*rqlH* and *rqlI* are co-expressed and are not regulated by oxygen levels. (A) A schematic of the *rqlHI* operon and surrounding genes, with the three primer sets used in (B) indicated. (B) RNA was isolated from F11 WT bacteria growing aerobically, and cDNA was made and used as template in PCRs using the three primer sets indicated in (A). Each primer set was used with a sample lacking reverse transcriptase (RT) as a control. Ladder band sizes are indicated at left. (C) F11 WT bacteria were grown either aerobically or microaerobically, RNA was isolated, and cDNA was made. Several primer sets were used in qRT-PCR reactions to measure levels of *frdA*, *rqlH*, *rqlI*, and *rqlHI* transcripts under the two oxygen conditions. All expression values were normalized to 16S rRNA. Bars indicate mean values ± SEM from three or more independent experiments performed in triplicate. *, *P* ≤ 0.05; as determined by Student’s *t* test.

In order to understand why *sulA* expression is increased in the Δ*rqlI* strain, a screen was designed to search for factors that promote SOS induction in the absence of RqlI (Fig 3A). An F11Δ*rqlI*Δ*lacZY* strain was transformed with plasmid pCWR2 containing the *lacZ* gene driven by the *sulA* promoter, providing a convenient readout of *sulA* expression levels on tetrazolium lactose plates. On these plates, high LacZ expression—reflecting high *sulA* promoter activity—results in colonies with pink to white color, while low LacZ activity due to low *sulA* promoter activity results in a red colony color. F11Δ*rqlI*Δ*lacZY/*pCWR2 was randomly mutagenized using the *mariner* transposon from pSAM_Ec [14], and then plated onto selective tetrazolium lactose plates. Approximately 20,000 colonies were screened for darker colony color, indicative of a decreased SOS response. Transposon insertion locations were mapped in potential suppressor mutants, and six independent insertions were identified within *rqlH* (Fig 3B). This gene encodes a homologue of the *Mycobacterium smegmatis* RqlH protein (MSMEG_5935), a RecQ-Like Helicase that is known to unwind dsDNA in a 3’ to 5’ orientation [21]. The F11 and *M*. *smegmatis* RqlH proteins align along their entire lengths, with 41.2% identity (55.7% similarity) overall (S2 Fig). Interestingly—and perhaps, in retrospect, not surprisingly—the F11 *rqlH* gene is also found adjacent to *rqlI* on the genome (Fig 4A). To confirm the results of the screen, *rqlH* and *rqlI* were deleted from F11, separately and in combination, and *P*_*sulA*_ *-GFP* expression levels were measured under low oxygen conditions (Fig 3C). As before, GFP expression levels in the Δ*rqlI* strain were higher than in WT F11, whereas strains lacking only *rqlH* or both *rqlH* and *rqlI* had GFP levels equal to that of WT. These data indicate that in the absence of RqlI, RqlH can trigger the SOS response, resulting in increased *sulA* expression.

To test whether the reduction in growth of F11Δ*rqlI* under microaerobic conditions could also be attributed to RqlH activity, WT F11 and the Δ*rqlI*, Δ*rqlH*, and Δ*rqlHI* strains were grown in low oxygen cultures. Relative to the WT strain, only the Δ*rqlI* mutant grew poorly in these assays (Fig 3D). In addition, we found that overexpression of RqlH caused a marked reduction in bacterial growth under both aerobic and microaerobic conditions (S3 Fig). This effect was seen regardless of genetic background, but was more pronounced in strains that lacked RqlI. Together, these results indicate that RqlH activity in the absence of RqlI both induces the SOS response and inhibits bacterial growth.

### RqlH increases bacterial sensitivity to DNA damage in the absence of RqlI

As the SOS response is activated by DNA damage and is responsible for initiating DNA repair programs, we examined the abilities of the *rqlI* and *rqlH* mutants to deal with exogenous DNA damage. WT F11 and mutant derivatives were treated with the DNA crosslinking agent mitomycin C and surviving bacterial cells were quantified. The Δ*rqlI* mutant had increased sensitivity to mitomycin C, a defect that was again rescued in the Δ*rqlHI* double mutant strain (Fig 3E) and by complementation with the RqlI expression plasmid pCWR16 (Fig 3F). A similar trend was observed when the cells were treated with UV light. The Δ*rqlI* mutant was more sensitive to both 25 and 50 J/m^2^ of UV treatment, although this effect was only statistically significant at 25 J/m^2^ (Fig 3G). In contrast, the Δ*rqlH* and Δ*rqlHI* mutants survived UV exposure at levels similar to the WT cells. These data indicate that the Δ*rqlI* mutant is more sensitive to DNA damage in a manner that is dependent on RqlH.

To determine if the increased sensitivity of the Δ*rqlI* mutant to exogenous DNA damaging agents correlates with a decreased ability to deal with endogenous DNA damage, spontaneous mutation rates that resulted in nalidixic acid resistance were quantified using fluctuation assays [22]. WT F11 and the Δ*rqlI*, Δ*rqlH* and Δ*rqlHI* strains all exhibited low mutation rates of about 3 mutations per 10^10^ cells per generation when grown under aerobic conditions (Fig 3H). Growth under microaerobic conditions did not change the mutation rates for WT F11, F11Δ*rqlH*, or F11Δ*rqlHI*, but resulted in nearly an 80-fold increase in the mutation rate of the Δ*rqlI* mutant. These data suggest that in the absence of RqlI, RqlH makes ExPEC more sensitive to DNA damage and the accumulation of spontaneous mutations.

### *rqlH* and *rqlI* are co-expressed in an oxygen-insensitive fashion

Since the *rqlH* and *rqlI* genes are located immediately adjacent to each other on the genome, but are separated from upstream and downstream genes, it is likely that they form a two-gene operon (Fig 4A). To test this idea, we performed RT-PCR using primer sets spanning the *rqlH* and *rqlI* loci, as well as one set internal to *rqlI*, as depicted in Fig 4A. Products expected if *rqlH* and *rqlI* are co-transcribed as part of the same transcript were obtained from F11 cDNA, but were not seen in control reactions that lacked reverse transcriptase (Fig 4B). These results indicate that *rqlH* and *rqlI* form an operon.

Given that the Δ*rqlI* mutant defect is exacerbated by microaerobic conditions, we tested whether *rqlHI* transcription is modulated by oxygen levels. Using primers that are specific for *rqlH*, for *rqlI*, or that span *rqlHI*, we were unable to detect any changes in *rqlHI* expression in microaerobic growth as compared to aerobic growth (Fig 4C). In contrast, we observed that transcription of *frdA*, which is sensitive to oxygen levels [23], was significantly upregulated in F11 grown under microaerobic conditions.

### The PRTase domain of RqlH is genotoxic in the absence of RqlI

**Fig 5:**
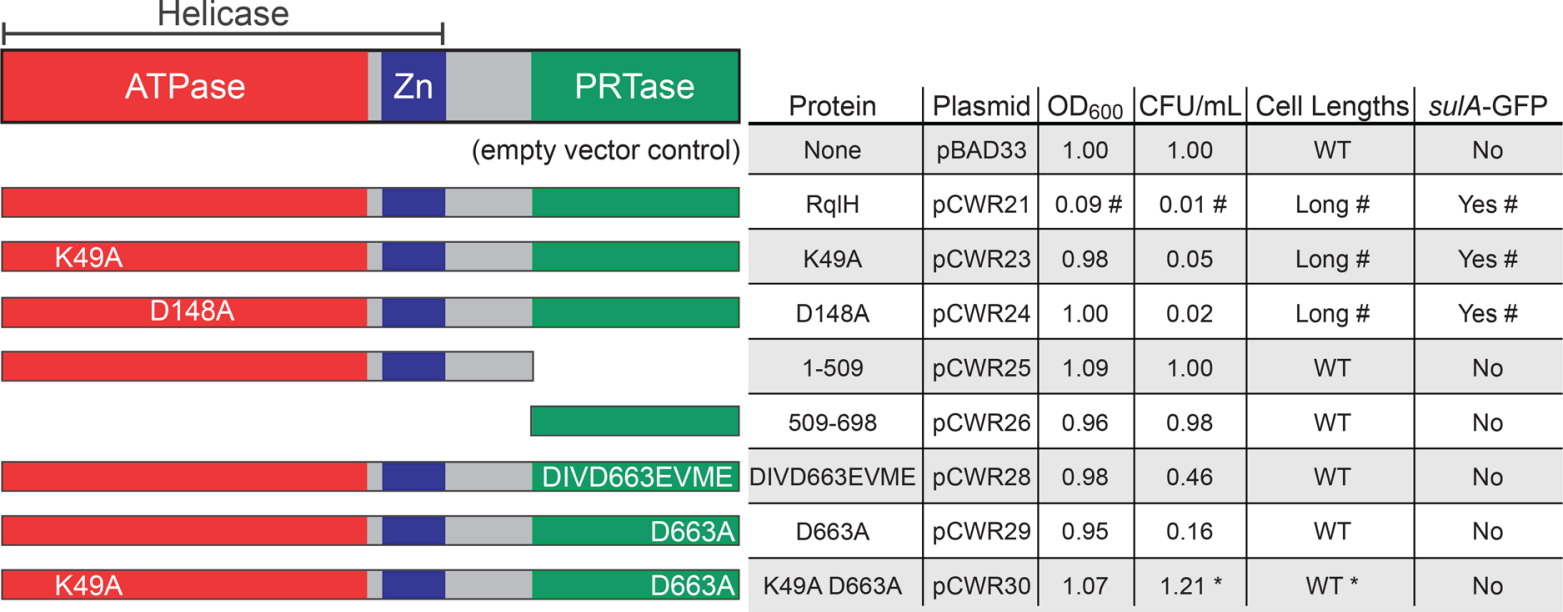
The RqlH PRTase domain is required for SOS induction in the absence of RqlI and RqlH-associated helicase activity. On the left are schematic representations of the domains within the F11 RqlH protein and mutant variants that were expressed in MG1655 cells. Table summarizes the effects of each RqlH expression construct on bacterial growth (OD_600_), viability (CFU/ml), cell length, and SOS induction (determined using the reporter strain MG1655 *attTn7*::P_*sulA*_-GFP). Results are from 6 h shaking cultures in modified M9 media, and are normalized to control bacteria carrying the empty vector pBAD33. #; phenotype is rescued by co-expression of RqlI. *; phenotype is exacerbated by co-expression of RqlI. See S5 and S6 Figs for full data.

RqlH contains an N-terminal helicase domain as well as a C-terminal phosphoribosyltransferase (PRTase) domain that is unique among known helicases [21]. The functional importance of the PRTase domain is unclear, though it is dispensable for the ATPase and helicase activities of RqlH in *M*. *smegmatis* [21]. To define domain(s) within RqlH that are responsible for the genotoxic effects observed in the absence of RqlI, we expressed epitope-tagged RqlH and several mutant variants in the K-12 strain MG1655, which lacks both RqlH and RqlI. Expression of each RqlH variant was confirmed by western blot (S4 Fig). The recombinant MG1655 strains were diluted into modified M9 minimal media and grown for 6 h with aeration, at which point the optical density (OD_600_) of each culture and the numbers of viable bacteria (CFU/mL) were measured. Expression of the WT RqlH significantly reduced both the growth of MG1655 and the numbers of CFUs recovered (Figs 5 and S5). RqlH expression also stimulated the formation of filamentous bacteria and the induction of the SOS response, as determined by microscopy and by use of the SOS reporter strain MG1655 *attTn7*::P_*sulA*_-GFP (Figs 5 and S6). As seen with F11, the toxic effects of RqlH expression in MG1655 were negated by co-expression of RqlI.

Deletion of the C-terminal PRTase domain (amino acids 509–690) of RqlH abrogated the growth-inhibitory and SOS-inducing effects of RqlH expression in the absence of RqlI (Figs 5, S5 and S6). To examine the role of the PRTase domain more specifically, residues within the putative phosphoribosylpyrophosphate binding pocket of RqlH were mutated. Two PRTase domain variants were tested: one having four conservative amino acid substitutions (DIVD663EVME; [24]) within the putative binding pocket and one having a single alanine replacement (D663A). These residues were chosen based on alignments of RqlH with two phosphoribosyltransferases, PurF and Hpt, and by overlaying the crystal structure of PurF with the predicted structure of RqlH (S7 Fig). Expression of either the DIVD663EVME or D663A RqlH mutant variants had no notable effects on bacterial growth, activation of the SOS response, or filamentation, and had only modest inhibitory effects on the numbers of viable bacteria recovered from the cultures (Figs 5, S5 and S6). These data indicate that the PRTase domain is necessary for RqlH-mediated toxicity, and therefore the defects associated with the Δ*rqlI* mutant. However, expression of the PRTase domain alone was not sufficient to inhibit bacterial growth and did not trigger activation of the SOS response or filamentation, suggesting that additional elements within RqlH cooperate with the PRTase domain to promote toxicity in the absence of RqlI (Fig 5).

To better understand the role of the helicase domain of RqlH, we tested two mutant proteins with single amino acid substitutions (K49A and D148A) that were previously shown to abrogate RqlH helicase activity in *M*. *smegmatis* [21]. When these mutant proteins were expressed in MG1655, the bacteria grew to a normal density, but low CFU counts were recovered from the cultures (Figs 5 and S5). This reduction in CFUs coincided with increased activation of the SOS response and the development of more filamentous cells by the K49A and D148A mutants (Figs 5 and S6). The loss of viable CFUs was not rescued by co-expression of RqlI, though RqlI did diminish the effects of the K49A and D148A mutations on activation of the SOS response and filamentation. Titers were restored to WT levels if RqlH carried both the K49A mutation within the helicase domain and the D663A mutation within the PRTase domain (Figs 5 and S5). These data suggest that both RqlI and the helicase domain of RqlH are required to keep the PRTase domain of RqlH in check. However, it is clear that these regulatory interactions are complex. As a case in point, the combined expression of RqlI with the double mutant RqlH K49A/D663A protein leads to a reduction in bacterial titers without a coordinate decrease in culture density, as seen with the single K49A and D148A RqlH single mutants. (Figs 5 and S5). These data are further complicated by the fact that mutations within helicases often have dominant-negative effects that disrupt DNA metabolism [25].

The discordance between culture densities and bacterial CFUs observed with recombinant strains that express the K49A or D148A RqlH mutants ± RqlI, or with bacteria that express both RqlI and the RqlH K49A/D663A double mutant (S5 Fig), is likely in part attributable to stress-induced inhibition of septation and the formation of filamentous cells. Filamentous bacteria will increase the optical density of a culture, but may register as fewer CFUs in plating assays. In our experiments, recombinant strains that reached WT culture densities but had much less than WT titers tended to have more filamentous bacteria (Figs 5, S5 and S6). For the K49A and D148A mutants, filamentation in the absence of RqlI correlated with activation of the SOS response, but this phenomenon was notably less pronounced when RqlI was also expressed (S6 Fig). The co-expression of RqlI with the K49A/D663A RqlH mutant protein had little effect on SOS activation, even though filamentation levels were elevated and CFU counts were markedly reduced. These data indicate that SOS-dependent and SOS-independent filamentation may contribute to the reduced CFU counts recovered following growth of strains that express RqlH helicase domain mutants.

The discrepancies between cell culture densities and titers seen with some of the recombinant strains could also be explained by the generation of anucleate cells or by plating deficiencies, whereby cells may grow well in broth culture but poorly when spread onto agar. To test the former possibility, bacteria were stained using the fluorescent DNA dye Hoechst and imaged. By microscopy, no cells that lack DNA were detected among any of the recombinant strains examined (see S8 Fig as an example). To test for plating deficiencies, bacteria were diluted into fresh LB broth culture at the end of a 6 h growth period and the OD_600_ was tracked over time. Those strains that had decreased CFU counts at the end of the initial 6 h incubation also had a lag in growth when sub-cultured 1:100 into fresh broth, as indicated in S5 Fig as an increase in the time required to reach an OD_600_ of 0.4. These results suggest that the recombinant strains that reach WT density levels in culture, but have reduced titers on agar, have fewer viable cells and have no inherent plating deficiencies.

### Overlapping domains within RqlI counter the toxicity of RqlH

**Fig 6:**
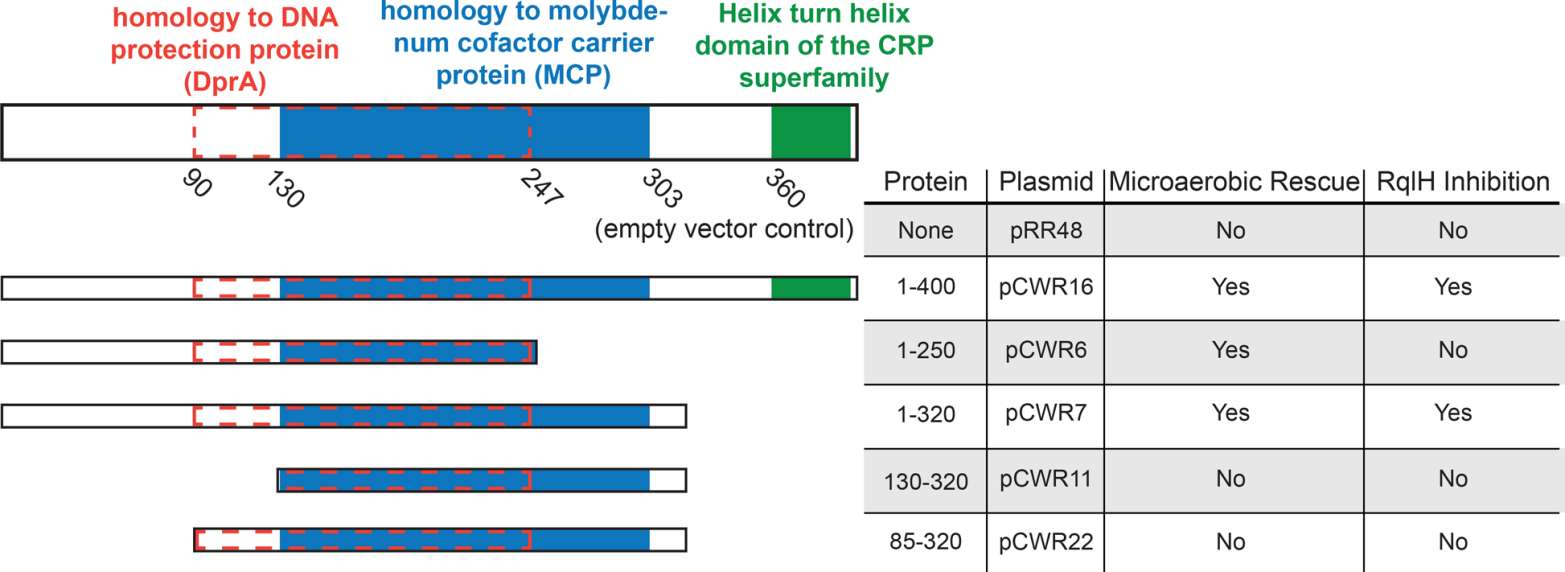
Domains within the N-terminus of RqlI counter the toxicity of RqlH, independent of the putative HTH domain. The schematics on the left indicate the locations of predicted domains and homology regions within RqlI and various truncation mutants. The table summarizes the effects of each RqlI expression construct on F11Δ*rqlI* titers recovered from 24 h cultures grown microaerobically. Whether or not expression of a given RqlI variant significantly increased bacterial titers in comparison to the empty vector control is indicated under “Microaerobic Rescue”. The “RqlH Inhibition” column indicates if a given RqlI variant could counter the toxic effects of RqlH when expressed in MG1655 grown for 6 h under aerobic conditions. See S10 Fig for full datasets.

As already noted, RqlI contains three putative domains, including a region of homology to DprA, a MoCo binding domain, and a helix-turn-helix (HTH) domain (Fig 1A). To test whether these various domains are required for RqlI function, plasmids encoding different truncation variants of RqlI were created and transformed into F11Δ*rqlI*. Western blots indicated that the recombinant RqlI proteins were expressed, though at varying levels (S9 Fig). As shown in Fig 1B, the growth defect observed with F11Δ*rqlI* under microaerobic conditions can be partially rescued by expression of the full-length RqlI protein (1–400; pCWR16 in Figs 6 and S10A). Expression of the N-terminus of RqlI plus the DprA homology region (1–250; pCWR6) also partially rescued growth of F11Δ*rqlI*. Of note, the 1–250 RqlI mutant complemented F11Δ*rqlI* much like the full-length RqlI even though relatively low levels of the truncated protein were detected (S9 Fig). Nearly complete rescue of F11Δ*rqlI* was attained by expression of an RqlI truncation mutant (1–320) that lacked the C-terminal 80 residues, including the putative HTH domain (Figs 6 and S10A). These results indicate that the HTH domain is not needed for full RqlI activity under microaerobic conditions, and that the HTH domain may instead actually be somewhat inhibitory to bacterial growth in these assays. In contrast, the expression of RqlI deletion mutants that lack the HTH domain as well as the N-terminal 129 or 84 residues adjacent to the MoCo binding and DprA homology domains did not complement F11Δ*rqlI*.

We also examined the ability of the RqlI variants to abrogate the inhibitory effects of RqlH expression on growth of MG1655 under aerobic conditions (Figs 6 and S10B). Paralleling results obtained with F11Δ*rqlI*, the expression of RqlI truncation mutants that lack the HTH domain as well as the N-terminal 84 or 129 amino acids were unable to counter RqlH, while the production of full-length RqlI or the 1–320 truncation mutant effectively rescued growth of RqlH-expressing MG1655. In contrast, the 1–250 RqlI truncation mutant that restored growth of the F11Δ*rqlI* strain under microaerobic conditions was unable to block RqlH-mediated toxicity in MG1655 under aerobic conditions. In total, these results indicate that the HTH domain is dispensable to the ability of RqlI to interfere with the toxic effects of RqlH under either aerobic or microaerobic conditions. It is also clear that the N-terminus, including both the DprA and MoCo binding domains, is required for full RqlI activity in these assays. However, the C-terminal portion of the putative MoCo binding domain is differentially required, potentially dependent on strain background and/or oxygen levels.

### The RqlH and RqlI proteins interact

**Fig 7:**
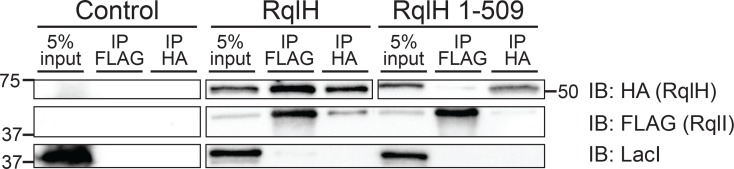
RqlH and RqlI proteins physically interact. MG1655 bacteria that express full-length FLAG-tagged RqlI and HA-tagged RqlH (either full-length RqlH or the 1–509 truncation mutant lacking the PRTase domain) were grown for 4 h, then an additional hour in the presence of IPTG and arabinose, prior to lysis under non-denaturing conditions. As a control, MG1655 carrying the empty vectors pRR48 and pBAD33 were processed in parallel. Lysates were immunoprecipitated using anti-FLAG or anti-HA antibodies, and immunoprecipitates were resolved by SDS-PAGE in preparation for western blot analysis. Aliquots (5%) of the cell lysates that were used as input for the immunoprecipitations were also resolved. Immunoblots (IB) were probed using antibodies specific for the FLAG or HA epitopes or for LacI, as indicated.

To test whether the ability of RqlI to keep RqlH activity in check involves physical interaction between the two proteins, co-immunoprecipitations were performed using MG1655 expressing FLAG-tagged RqlI and HA-tagged variants of either full-length RqlH or a C-terminal truncation mutant (1–509) that lacks the PRTase domain (Fig 7). As expected, the RqlH and RqlI proteins were not detected in the control strain carrying the empty plasmid constructs. Full-length RqlH was co-immunoprecipitated with RqlI, and vice-versa, suggesting that these two proteins do associate. This interaction was abrogated in bacteria that express the RqlH 1–509 truncation mutant, indicating that RqlI likely binds the PRTase domain of RqlH. As a control for the specificity of the co-immunoprecipitation procedure, we note that the *lac* operon repressor protein LacI, which is not expected to interact with either RqlH or RqlI, was not pulled down in these assays.

### RqlI is required for ExPEC persistence within the gut

**Fig 8:**
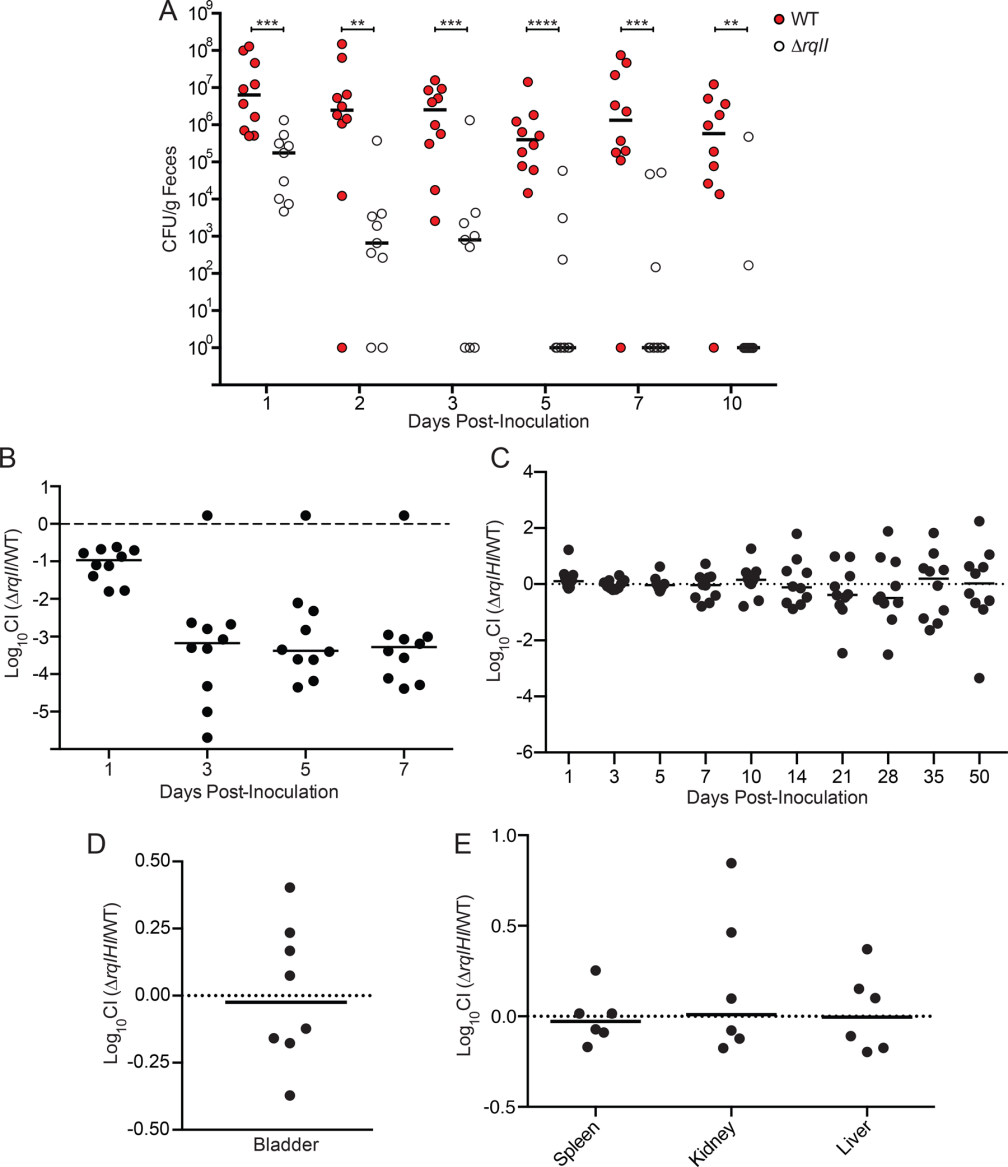
Decreased fitness of Δ*rqlI* mutants within diverse host niches is attributable to RqlH. (A) WT F11 or F11Δ*rqlI* were inoculated into Balb/c mice via oral gavage and feces were collected, homogenized, and plated to determine bacterial titers at the indicated time points. For competitive assays, (B) F11 and F11Δ*rqlI* or (C) F11 and F11Δ*rqlHI* were mixed 1:1 prior to delivery into mice via oral gavage. Graphs show competitive indices (CI), as determined by dilution plating of fecal homogenates on selective media at the indicated time points. (D) F11 and F11Δ*rqlHI* were inoculated transurethrally as a 1:1 mixture into the bladders of Balb/c. CI values were calculated from bacterial titers recovered from bladders at 3 d post-inoculation. (E) F11 and F11Δ*rqlHI* were mixed 1:1 and injected intraperitoneally into mice to initiate systemic infections. At 12 h post-inoculation, the spleen, liver, and a kidney were isolated from each mouse, homogenized, and titered to determine the CI of the Δ*rqlHI* mutant. Bars indicate median values.

Our finding that RqlI is an especially important regulator of RqlH toxicity under low oxygen conditions (Fig 3D) fits well with previous work in which we showed that RqlI is critical to ExPEC survival within the urinary tract and bloodstream [14]. These niches, like many sites of infection, have limiting amounts of oxygen available for use by facultative anaerobes like ExPEC. Since a primary reservoir of ExPEC within vertebrate hosts is the intestinal tract, in which oxygen levels are generally low [26], we set out to determine if RqlI was also important to the fitness of F11 within the gut. For these studies, we used marked strains that carried chromosomal Clm^R^ or Kan^R^ cassettes. Adult Balb/c mice were inoculated via oral gavage with 10^9^ CFU of F11 or F11Δ*rqlI*, and survival was tracked by titration of fecal pellets on selective plates. WT F11 persisted within the gut at fairly steady levels (medians ~10^6^ CFU/g feces) for more than 10 d, while titers of the Δ*rqlI* mutant were significantly reduced as early as day 1 (Fig 8A). By the end of the experiment, only 2 of the 10 mice that received F11Δ*rqlI* remained colonized with the mutant. The persistence defect observed with F11Δ*rqlI* was also evident when the mutant was co-inoculated 1:1 with WT F11. In these competitive assays, the WT strain outnumbered the Δ*rqlI* mutant by more than a 1,000-fold within 3 d (Fig 8B).

To test if RqlI is important for gut colonization by other ExPEC strains, *rqlI* was deleted from the urosepsis isolate CFT073 and from the pyelonephritis isolate 536. In competition assays, CFT073Δ*rqlI* titers within the intestinal tract were greatly diminished within 3 d of inoculation, mirroring the situation seen with F11 (S11A Fig). While 536Δ*rqlI* was also defective in gut colonization, the decline of this mutant was more gradual and less pronounced, decreasing about a 100-fold relative to the WT 536 strain by day 7 post-inoculation (S11B Fig). These results, coupled with our previous findings [14], indicate that RqlI promotes the colonization and persistence of various ExPEC strains within distinct host environments, including the urinary tract, the bloodstream, and the intestinal tract.

To determine if the *in vivo* defects observed with the Δ*rqlI* mutants are attributable to the disregulation of RqlH activities, as seen in our *in vitro* assays, F11 with a complete deletion of the *rqlHI* operon was competed 1:1 against the WT F11 strain in our mouse models for gut colonization, UTI, and bacteremia. Following oral inoculation of Balb/c mice with a 1:1 mixture of F11 and F11Δ*rqlHI*, fecal bacterial titers were tracked over the course of 50 days (Fig 8C). Median competitive indices stayed close to zero throughout the experiment, indicating that the Δ*rqlHI* mutant does not have a defect in mouse gut colonization, in sharp contrast to the single Δ*rqlI* mutant. Defects previously observed with F11Δ*rqlI* survival within the bladder and the bloodstream of mice [14] were also ablated if *rqlH* was deleted together with *rqlI* (Fig 8D and 8E). In total, these data indicate that the toxicity associated with RqlH expression in the absence of RqlI in our *in vitro* assays is also manifest *in vivo* within diverse host environments. Although the *in vivo* defects associated with the *rqlI* mutant are not particularly surprising in light of our *in vitro* findings, it is noteworthy that the *in vivo* effects are generally greater in magnitude than those seen *in vitro*.

### RqlH and RqlI orthologs are found across multiple genera, but are not always linked

Given the close relationship between *rqlH* and *rqlI* that we observed in F11, we wondered if these proteins are always present as a pair within bacterial genomes, if they are always adjacent to one another, and if they are widely distributed among bacteria. A search for orthologs of RqlH and RqlI was undertaken using NCBI sequence databases, followed by analysis of the results using custom Python scripts. We defined an ortholog as a hit that aligns with at least 75% of the RqlI or RqlH protein sequence and that has a percent positive score of at least 50 and an E-value of 1e-06 or better. In addition, putative orthologs had to successfully return the original query protein (RqlI or RqlH) as the top hit in reciprocal BLAST searches against F11. For a list of RqlI and RqlH orthologs, and for the raw data used in our analysis, see S1 Dataset.

**Fig 9:**
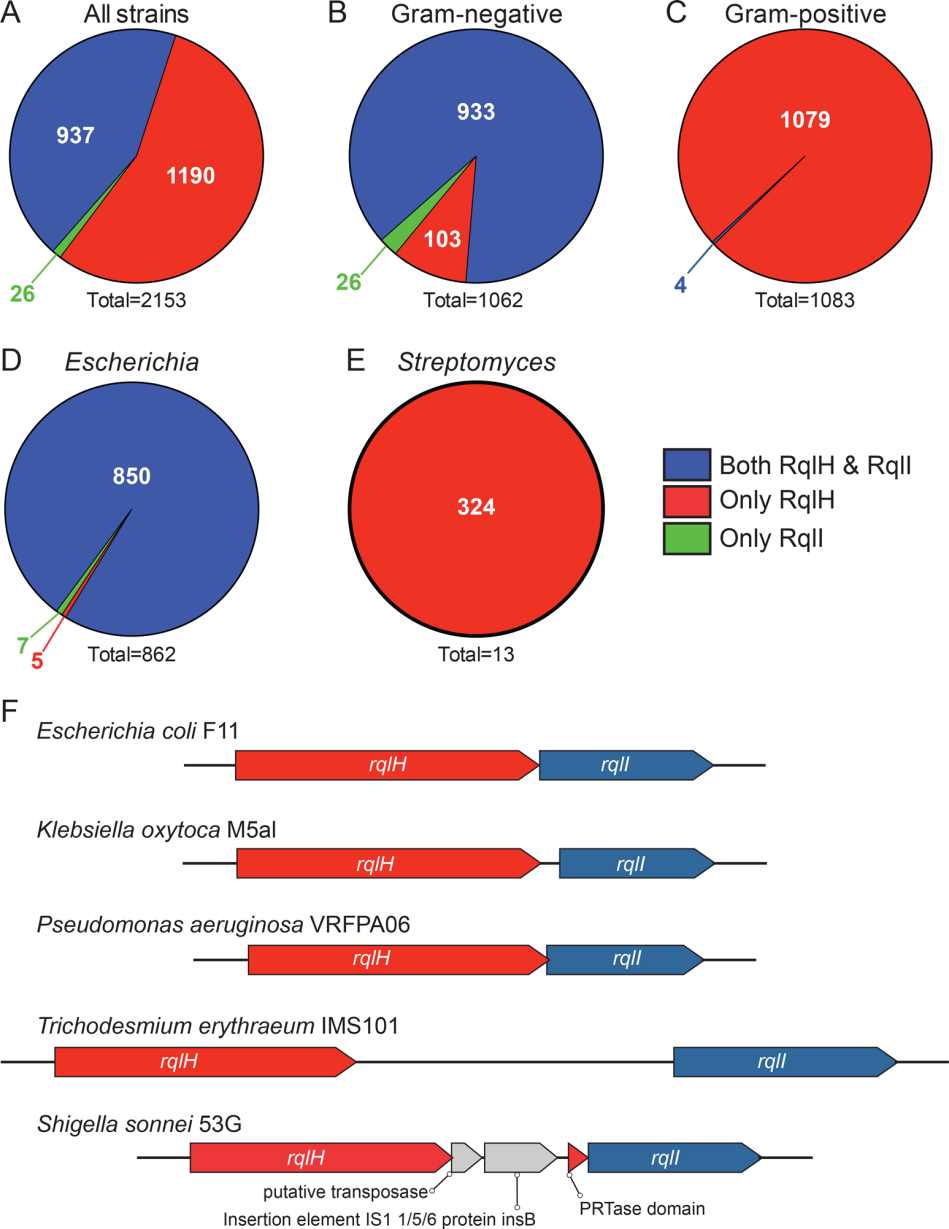
RqlH and RqlI homologs are found across various genera, either alone or together. F11 RqlH and RqlI homologs were found using blastp and tblastn searches of various NCBI sequence databases. (A-E) Distribution of strains containing only a homolog of RqlI (green), only RqlH (red), or both RqlH and RqlI (blue). The numbers of bacterial strains that are associated with each pie section are indicated, with totals given beneath each graph. Included are (A) distributions of RqlH and RqlI orthologs in all strains, (B) among Gram-negative bacteria, (C) among Gram-positive organisms, (D) within the *Escherichia* genus, or (E) as part of the *Streptomyces* genus. (F) Examples showing the arrangements of *rqlH* and *rqlI* orthologs in different bacterial species.

Our results indicate that RqlH or RqlI orthologs are distributed across a phylogenetically diverse range of bacteria, with a total of 240 distinct genera represented in the analysis. Unexpectedly, the majority of bacterial strains (55.3%) encoded an ortholog of RqlH, but had no detectable ortholog of RqlI (Fig 9A). There was also a small portion (1.2%) of the bacteria that contained only RqlI. 43.5% of bacteria within the database encoded both RqlI and RqlH.

By analyzing different bacterial subsets, we found that there is a striking difference in the distribution of RqlH and RqlI orthologs in Gram-positive versus Gram-negative. The majority (87.9%) of Gram-negative bacteria encode both RqlI and RqlH, with just 9.7% carrying only RqlH (Fig 9B). In contrast, nearly all (99.6%) Gram-positive bacteria encode only RqlH, while the remaining few have both proteins (Fig 9C). Bacteria with only an RqlI ortholog were found exclusively among the Gram-negative bacteria (Fig 9B). These observations were echoed in our analysis of specific individual genera. For example, 98.6% of the *Escherichia* strains (Gram-negative) encode both RqlH and RqlI (Fig 9D), while very few have only RqlH or only RqlI. *Streptomyces* species—like most other Gram-positive taxa—encode only RqlH, with no detectable orthologs of RqlI in any of the strains (Fig 9E). This is also the case for all strains of *Mycobacterium smegmatis*, the Gram-positive acid-fast species in which RqlH was first identified [21].

Within strains that have both *rqlH* and *rqlI* orthologs, we found that the genes are always oriented with *rqlH* upstream of *rqlI*, reminiscent of the operon structure in F11. In most of these strains, the start of *rqlI* is close to or overlapping with the terminus of *rqlH*, though in some strains the two genes are separated by more than 2 kbp. Fig 9F shows examples of the variable arrangements of *rqlH* and *rqlI* within divergent species. In *Klebsiella oxytoca* M5aI and *Pseudomonas aeruginosa* VRFPA06, the *rqlH* and *rqlI* genes are closely associated, or even slightly overlapping, whereas within the cyanobacterium *Trichodesmium erythraeum* IMS101 the two genes are separated by an unannotated 2196 bp sequence. Occasionally, we identified other annotated elements inserted between the *rqlH* and *rqlI* loci, as in *Shigella sonnei* 53G where sequences encoding the PRTase domain of RqlH have been separated from the helicase domain by an insertion element and a putative transposase. Together, these observations demonstrate that *rqlH* and *rqlI* orthologs, when found together within the same genome, are largely syntenic with the F11 *rqlHI* operon. The exceptions to this arrangement, as in *Shigella sonnei* 53G, may reflect alternate ways in which bacteria have evolved to modulate the potentially toxic effects of RqlH expression.

## Discussion

**Fig 10:**
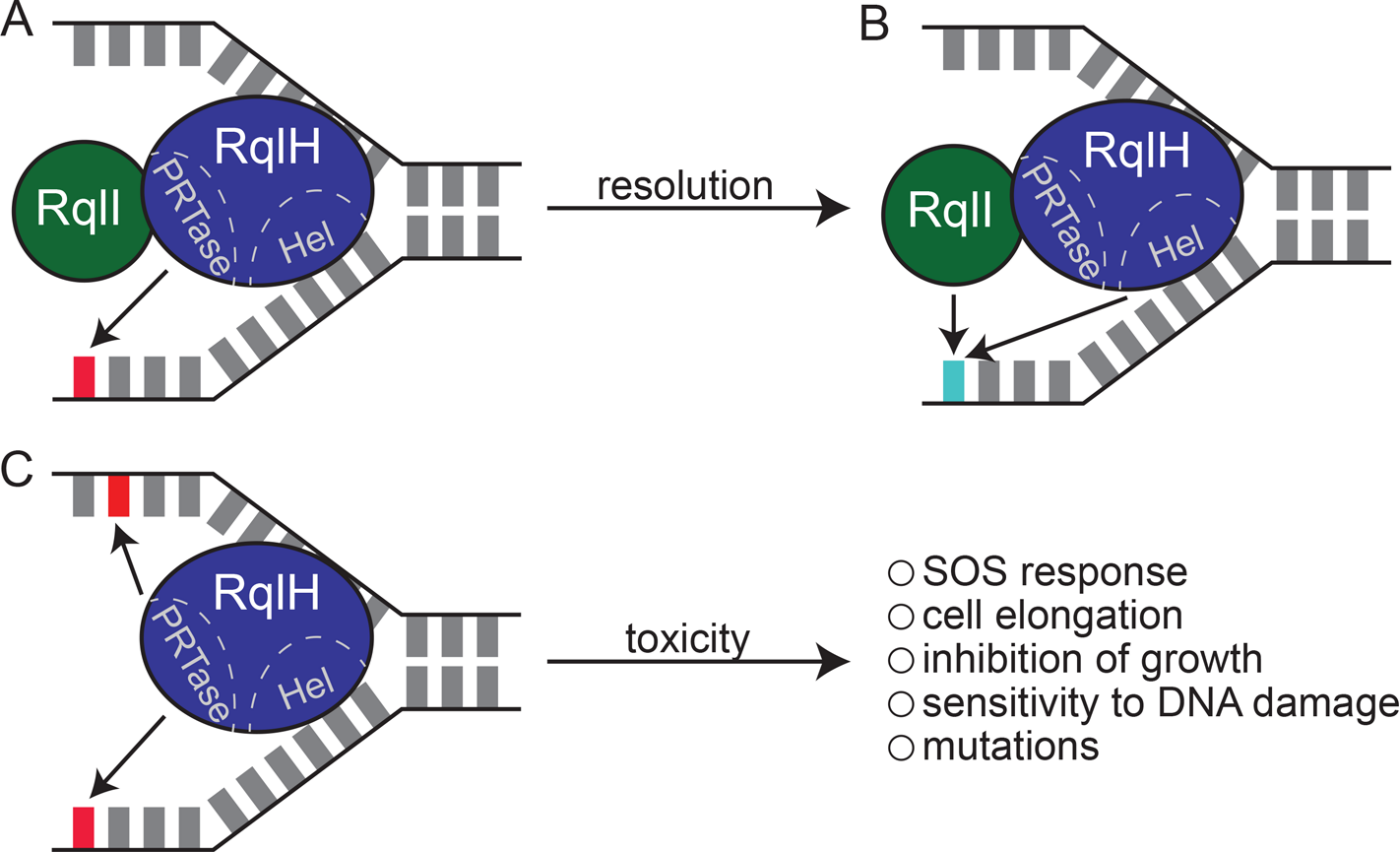
In the absence of functional RqlI the PRTase domain of RqlH elicits genotoxic effects. Model depicts a possible function for the *rqlHI* operon. (A) The PRTase domain of RqlH alters a nucleotide (red) within the bacterial genome, and (B) RqlI functions to resolve this modified nucleotide (light blue) with the help of the helicase domain of RqlH. The resolution event could include further modification of the nucleotide or restoring the nucleotide to its original structure. (C) In the absence of RqlI, the PRTase domain of RqlH continues to alter chromosomal nucleotides, but these modifications are not resolved, producing effects that are toxic to the bacteria, resulting in the induction of the SOS response, cell elongation, growth inhibition, heightened sensitivity to DNA damaging agents, and increased mutation rates. Many of these phenotypes are intensified under low oxygen conditions for unknown reasons.

This study was aimed at functionally defining *EcF11_3933*, a hypothetical gene that we previously identified as an important facilitator of ExPEC fitness in zebrafish infection models and in mouse models of UTI and sepsis [14]. Homologs of this gene are also expressed by UPEC isolates recovered directly from the urine of women with UTI [27]. Results presented here indicate that the *EcF11_3933* gene product, which we have renamed RqlI for RecQ-Like Helicase Interactor, is able to bind to and modulate the activity of the RecQ-Like Helicase RqlH. We propose a model in which RqlI and RqlH act cooperatively to perform an as-yet undefined beneficial function on bacterial DNA (Fig 10). Although more work is required to further vet this idea, the hypothesis is supported by several pieces of data. First, the RqlH homolog in *M*. *smegmatis* is known to be a helicase belonging to the RecQ family [21], which includes several helicases that perform general maintenance functions on prokaryotic and eukaryotic genomes [28]. Second, RqlH and RqlI physically interact (Fig 7) and are encoded within the same operon (Fig 4A and 4B). Third, the Δ*rqlI* mutant experiences several defects that are attributable to RqlH activities, all of which involve phenotypes associated with DNA stress. These include induction of the SOS response, increases in cell length (filamentation), higher sensitivity to DNA damaging agents, and elevated mutation rates (Figs 2 and 3). We suggest that in the presence of a partially functional or disregulated RqlH-RqlI system, some harmful DNA byproduct(s) is created that leads to a loss in cellular growth and viability (Fig 10B). Interestingly, the defects associated with disruption of the RqlH-RqlI system are exasperated under low oxygen conditions, suggesting possible links with anaerobic respiration and/or other redox-sensitive processes.

Our observations and the model presented in Fig 10 raise a number of questions concerning the RqlH-RqlI system. Chief among these is the question of function. We were unable to detect any decrease in bacterial fitness in Δ*rqlHI* mutants in our *in vitro* experiments or in mouse models of infection, using either competitive or non-competitive assays (Figs 8 and S11). These results may reflect limitations in the resolution of the mouse models, or their inability to recapitulate all aspects of natural colonization and infection processes. For example, our assays employed human ExPEC isolates that may use different strategies to colonize the human versus the murine intestinal tract. The intestinal microbiota associated with humans and mice are often similar at the phyla level, but can be quite different qualitatively and quantitatively at lower taxonomic levels [29,30]. Such differences may affect the types of nutrients and stresses encountered by ExPEC within the gut and could potentially influence the requirements for genes like *rqlHI*. ExPEC are versatile organisms, and so it is also feasible that RqlH-RqlI are important to the survival of these pathogens within environmental reservoirs or at other sites that were not modeled in our study.

The key to understanding the role of the RqlH-RqlI system in bacterial physiology may be the PRTase domain of RqlH. Indeed, RqlH is unique among helicases because it possesses a PRTase domain in addition to its helicase domain. PRTase domains can be found in proteins that function in nucleotide salvage pathways by adding phosphoribosyl groups to spent nucleotides. As an example, the guanine-xanthine phosphoribosyltransferase (Gpt) of *E*. *coli* catalyzes the addition of phosphoribosyl pyrophosphate (PRPP) to guanine to make the nucleotide guanosine monophosphate (GMP) [31]. Given the combination of a helicase with a PRTase domain in a single protein, it is tempting to speculate that RqlH functions to add PRPP directly to DNA, or that it adds PRPP to nucleobases while in association with DNA.

The function of *rqlHI* may be related to uptake of foreign DNA, as there are at least two connections between the RqlH-RqlI system and natural transformation in competent bacteria. First, RqlI contains a region of homology to DprA, a protein that has been shown to protect ssDNA during the translocation of foreign DNA into naturally competent bacteria [32]. Of note, in addition to the DrpA homology region within RqlI, F11 and other ExPEC isolates encode a DprA protein that is more closely related to the canonical DprA. However, DprA has no detectable effect on transformation in a K-12 *E*. *coli* strain [33]. The second connection to natural transformation is the PRTase domain of RqlH, a domain that is found in a number of proteins involved in competence in other bacteria. In *Bacillus subtilis*, two proteins encoded by the *comF* operon are suggestive of the functional domains contained within RqlH. Specifically, the *comF* operon, which *B*. *subtilis* requires for competence, codes for a putative helicase and a predicted PRTase separated by an intervening gene with no known domains [34]. The Com cluster of genes that facilitate transformation in *Haemophilus influenzae* also includes a gene (ComF) that is homologous to the PRTase domain of RqlH, but it is seemingly not associated with any nearby helicase [35]. Arguing against a role of the *rqlHI* operon in natural transformation is the fact that it is generally thought that *E*. *coli* is not naturally competent, though it can be made chemically competent or electrocompetent in a laboratory setting. Nonetheless, incidences of natural transformation by *E*. *coli* have been reported [36,37], but we have been unable to detect any transformation events using our ExPEC isolates, in agreement with a recent report [38].

In light of our results showing that RqlH is toxic to *E*. *coli* strains in the absence of RqlI (Fig 3), it was surprising to find that many bacteria carry RqlH but do not encode RqlI homologs (Fig 9). In particular, nearly all Gram-positive organisms that encode an RqlH homolog lack any identifiable RqlI ortholog (Fig 9C). These observations may be explained in several ways. It could be that RqlH on its own is not inherently toxic to some bacteria (e.g. Gram-positive microbes) due to differences in their physiology in comparison with ExPEC and the K12 strain MG1655. It is also possible that bacteria that lack *rqlI* express different, less toxic *rqlH* alleles than found in bacteria that encode both genes. However, we did not detect major differences in the sequences of RqlH proteins from bacteria that have just RqlH versus those with both RqlH and RqlI. Finally, RqlH activity could be kept in check by other proteins that are more distantly related to RqlI. In this same vein, it is interesting to note that RqlI orthologs are more prevalent among known pathogens (83.3% of RqlI homologs are encoded by pathogens in the TEA proteomic database; see [14]), while RqlH orthologs are much less often pathogen-associated (39.1%). Altogether, these observations suggest that RqlH function could vary depending on bacterial strain background and the presence or absence of RqlI.

The observation that RqlH expression in the absence of RqlI is toxic to *E*. *coli* strains is reminiscent of a type II toxin-antitoxin (TA) system. These TA systems consist of a protein toxin that is more stable than its cognate antitoxin protein, which binds to and inhibits the toxin [39]. TA systems were originally found encoded on plasmids where they promoted plasmid maintenance. In any cell that spontaneously loses the plasmid, the TA system is no longer transcribed, and the remaining toxin protein outlasts the residual antitoxin, resulting in bacterial cell stasis or death. In this and other similar situations, TA systems can be thought of as selfish genetic elements that function only to proliferate without providing any benefit to the host bacterium. More recently, TA systems have also been shown to have beneficial functions that can protect bacteria from stressors. For example, TA systems can promote the formation of antibiotic-insensitive persister cells [39] and select TA systems in ExPEC can promote stress resistance and colonization of the urinary tract [40].

Results presented here indicate that RqlH and RqlI can in many ways act like a TA system, meshing with data from others showing that an RqlH ortholog (PsyrT) in *Pseudomonas syringae* is toxic to the bacteria in the absence of the RqlI ortholog PsyrA [41]. However, we note that if RqlH and RqlI are to be defined as a TA system, it would be non-canonical for the following reasons: 1) RqlH and RqlI are much larger (698 and 400 amino acids, respectively) than typical TA system proteins (~100 amino acids); 2) *rqlH* is situated upstream of *rqlI*, whereas in most type II TA systems the antitoxin is encoded upstream of the toxin; and 3) an Δ*rqlHI* mutant exhibits increased survival (persisters) in the face of antibiotic treatments (S12 Fig), in contrast to the results obtained when *bona fide* TA systems are deleted [40]. Finally, we suggest that the distribution among most bacteria of RqlH orthologs that are encoded in the absence of RqlI argues against the possibility that the RqlH-RqlI system always functions as a TA-like genetic element, although it can resemble one within ExPEC strains.

Cumulatively, this work illustrates the complexities of assigning function to hypothetical genes that are identified as fitness determinants in high-throughput screens, such as Tn-seq. The ability of RqlI to counter the oxygen-sensitive genotoxic effects associated with RqlH helps explain why ExPEC strains are dependent on RqlI expression within the microaerobic confines of the gut and other extraintestinal niches. Specifically, our work indicates that RqlI functions to modulate the context-dependent toxicity of the RqlH helicase and its PRTase domain, but opens up many more questions concerning the functional relevance of the RqlH-RqlI system among ExPEC strains and of the numerous other RqlH and RqlI orthologs identified among thousands of phylogenetically diverse species. Future analyses detailing the regulation and functionality of the RqlH-RqlI system and its many homologs may provide the basis for novel anti-bacterial therapeutics that can unleash the toxic potential of RqlH.

## Materials and Methods

### Ethics statement

Mice were handled in accordance with protocols approved by the Institutional Animal Care and Use Committee at the University of Utah (Protocol number 10–02014), following US federal guidelines indicated by the Office of Laboratory Animal Welfare (OLAW) and described in the Guide for the Care and Use of Laboratory Animals, 8th Edition.

### Bacterial strains, media, and plasmids

The ExPEC strains used in this study included the cystitis isolate F11, the urosepsis isolate CFT073 and the pyelonephritis isolate 536, as well as the K-12 strain MG1655 (S1 Table). Manipulations of chromosomal DNA, including both the generation of knockout strains and the knockin strain MG1655 *attTn7*::P_*sulA*_-GFP were achieved with lambda-Red-mediated recombination using plasmid pKM208 [42]. Knockout strains were produced using PCR products containing ~40 bp overhang regions with homology to target loci. To create the MG1655 *attTn7*::P_*sulA*_-GFP reporter strain, a *tetA-sacB* cassette was amplified from T-SACK cells [43], and combined via PCR with two 500 bp arms homologous to the *attTn7* site downstream of *glmS*. Next, the *tetA-sacB* cassette was replaced with the P_*sulA*_-GFP reporter from pJLJ3 with selection on Tet/SacB counter-selection agar, as described [43]. Primers used to create knockout and knockin constructs, as well as those used for mutant confirmation, are listed in S1 Table. All PCR products were purified with the DNA Clean & Concentrator-5 kit (Zymo Research, catalog #D4004). Where indicated, antibiotic resistance cassettes were removed using the flippase-expressing pCP20 plasmid, as described [44].

Bacteria used for genetic manipulations were grown in LB broth, whereas bacteria used for both the *in vivo* and *in vitro* experiments were grown in modified M9 media. The modified M9 media contained M9 salts (6 g/L Na_2_HPO_4_, 3 g/L KH_2_PO_4_, 1 g/L NH_4_Cl, and 0.5 g/L NaCl), as well as 1 mM MgSO_4_, 0.1 mM CaCl_2_, 0.1% glucose, 0.00125% nicotinic acid, 0.00165% thiamine, and 0.2% casein amino acids.

All plasmids used in this study are listed in S2 Table. For previously unpublished plasmids, the primers utilized in their creation are also indicated. RqlI expression vectors were created by inserting PCR products into the PstI and HindIII sites of pRR48, under control of an IPTG-inducible promoter [45]. RqlH expression vectors were made by insertion at the SacI and HindIII sites within pBAD33, downstream of an arabinose-inducible promoter [46]. Plasmids were generated using standard cloning techniques, the QuikChange II XL Site-Directed Mutagenesis Kit by Agilent Technologies, or by overlap extension PCR [47], as indicated in S2 Table.

### Colonization of mice with ExPEC

Female Balb/c mice were purchased from Jackson Labs and used between 7–8 weeks of age, and all experiments were performed in accordance with IACUC-approved protocols. All mouse experiments were repeated at least twice, and the total combined data from six or more animals is presented. Bacteria used to inoculate mice were grown statically at 37°C in 20 mL modified M9 in 250 mL Erlenmeyer flasks for 24 h, pelleted by spinning at 8000 r.c.f. for 8 min, washed, and resuspended in phosphate buffered saline (PBS). For competitive assays, WT and mutant bacteria were mixed in equal parts prior to inoculation. These experiments used bacteria carrying either a chloramphenicol or kanamycin resistance cassette, allowing them to be easily selected and distinguished from other bacteria within the microbiota, and enabling the facile tracking of individual WT and mutant strains in competitive experiments.

To assess intestinal colonization by the ExPEC strains and their mutant derivatives, mice were orally gavaged with 50 μl of a bacterial suspension containing 1x10^9^ CFU of bacteria in PBS. Fecal samples were collected at various time points post-gavage, homogenized in 1 mL 0.7% NaCl, and briefly spun to pellet any insoluble debris. Supernatants were then serially diluted and plated on LB agar containing chloramphenicol (10 μg/ml) or kanamycin (50 μg/ml). Fecal samples were also collected and plated prior to gavage to ensure that the resident microbiota did not include any culturable chloramphenicol- or kanamycin-resistant bacteria.

For the UTI model, mice were anesthetized using isofluorane inhalation and slowly inoculated via transurethral catheterization with 50 μl of a bacterial suspension containing ~1x10^8^ bacteria in PBS. After 3 d, the bladders were extracted and homogenized in PBS. Homogenates were then serially diluted and plated.

To initiate bacteremia/sepsis, mice were injected intraperitoneally with 200 μl of PBS containing ~1x10^7^ bacteria. The animals were monitored for signs of extreme morbidity and euthanized at 12 h post-inoculation. The livers, kidneys, and spleens were then recovered and homogenized in PBS. Bacterial titers present within the various tissues were quantified by plating serial dilutions of the homogenates.

### Bacterial growth and reporter assays

To quantify bacterial growth in various oxygen levels, bacteria were grown overnight in M9 in loose-capped tubes, then subcultured the following day 1:100 in M9. During the subculture stage, the bacteria were grown on a shaking incubator for 24 h in 12-well plates either in aerobic conditions or in a Mitsubishi AnaeroPack 2.5 L Rectangular Jar holding a AnaeroPack-Microaero sachet or AnaeroPack-Anaero sachet to model microaerobic and anaerobic conditions, respectively. To calculate generation time, the bacteria were titered at two time points during exponential phase.

To measure *sulA*-GFP expression in F11 bacteria carrying the pJLJ3 plasmid or the pJLJ1 empty vector control, bacteria were grown overnight in loose-capped tubes in M9 with kanamycin, then subcultured 1:100 into fresh M9 with kanamycin. Cells were then grown microaerobically and anaerobically for 24 h in 12-well plates, or aerobically for 4 h in loose-capped tubes. For the control conditions, mitomycin C was added to a final concentration of 0.25 μg/mL in aerobic cultures at 3 h, and then incubated for another hour. Both GFP fluorescence and the OD_600_ were measured using a BioTek Synergy H1 plate reader.

For experiments with the MG1655*attTn7*::P_*sulA*_-GFP strain carrying various RqlH mutant proteins, the cells were grown overnight in M9 with ampicillin and chloramphenicol in loose-capped tubes The bacteria were then subcultured 1:100 into M9 with ampicillin, chloramphenicol, IPTG (1 mM), and arabinose (0.05%), and incubated aerobically in loose-capped tubes for 6 h. A growth curve was performed by again subculturing cells after the 6 h growth period 1:100 into fresh LB. The OD_600_ was then read every 30 minutes by a Bioscreen C machine.

In the assays, 1 mM IPTG or 0.05% arabinose was added to the media to induce expression of RqlI, RqlH or their derivatives.

### DNA damage sensitivity assays

Following overnight growth from frozen stocks, F11, F11Δ*rqlH*, F11Δ*rqlI*, and F11Δ*rqlHI* were diluted 1:100 into modified M9 media and grown with shaking (aerobically) for 4 h in loose-capped tubes. After addition of mitomycin C (0.25 μg/mL), the incubations were continued for one more hour. Bacterial titers before and after mitomycin C treatment were determined by plating serial dilutions. Survival rates were calculated as the numbers of viable bacteria present after mitomycin C treatment as a percent of bacteria present prior to addition of the toxin.

To assess strain susceptibility to UV irradiation, bacteria were grown aerobically in loose-capped tubes from overnight cultures for 4 h at 37°C, then serially-diluted and spread onto LB plates in order to obtain ~100 cells per plate. Plates were then exposed to 0, 25, or 50 J/m^2^ UV light produced by a Stratalinker 1800. The plates were then incubated overnight and surviving bacteria quantified.

### Microscopy

To visualize bacteria, 5 μl of cells (~5x10^7^ cells) resuspended in PBS containing 1 μg/mL Hoechst dye were spread on a glass slide and incubated at room temperature until the PBS had evaporated. A drop of FluorSave Reagent was added to each slide and a coverslip was placed on top. Bacteria were imaged by fluorescence or phase-contrast microscopy using an Olympus BX51 microscope equipped with a 100X oil immersion objective and a QImaging QIClick Cooled CCD camera. To measure cell lengths, straight-line measurements from one tip of a cell to the other were made using ImageJ [48]. For each bacterial strain, 6–10 fields and more than 200 bacteria were examined.

### Transposon screen

A P_*sulA*_-*lacZ* reporter plasmid (pCWR2) was created by overlap extension PCR using a promoterless *lacZ* plasmid (pCWR1) as a template (S2 Table). The reporter plasmid was introduced into F11Δ*rqlI*Δ*lacZY*::*clm*, and the resulting strain was randomly mutagenized by conjugation with EcS17/pSAM-Ec donor bacteria as described [14]. For conjugation, bacteria were grown overnight in LB broth, and 1 mL of donor cells were mixed with 0.5 mL of recipient cells, washed once with LB broth, and then spread onto an LB plate. After a 5 h incubation at 37°C, bacteria were recovered from the plate surface, resuspended in 1 mL of LB broth, and diluted 1:100. Aliquots (100 μl) were plated onto tetrazolium lactose plates containing ampicillin, chloramphenicol, and kanamycin to select for transposon-mutagenized F11Δ*rqlI*Δ*lacZY*::*clm*/pCWR2 bacteria. Colonies that were darker than the F11Δ*rqlI* Δ*lacZY*::*clm*/pCWR2 strain were re-streaked onto new tetrazolium lactose plates to verify decreased P_*sulA*_ *-lacZ* activity. Transposon mutants that returned *sulA* expression to WT levels underwent a secondary screen in a broth-based modified Miller assay, as described [49]. For mutants that exhibited decreased *sulA* expression on both tetrazolium lactose plates and in the Miller assays, transposon insertion sites were mapped by arbitrary PCR. Briefly, 30 μl colony PCR reactions were carried out using the pSAM-EC kan us1 primer paired with each of the arbitrary primers ARB1A, ARB1B, and ARB1C (S3 Table). The thermocycler program for this reaction was as follows: 95°C for 5 min; 5 cycles of 94°C for 30 sec, 30°C for 30 sec, and 72°C for 1:30; 30 cycles of 94°C for 30 sec, 45°C for 30 sec, and 72°C for 2 min; and ending with a 5 min incubation at 72°C. A 2 μl aliquot of this reaction was then used as template in a second PCR reaction using the pSAM-EC kan us2 and ARB2 primers, in a total volume of 50 μl. The second thermocycler program was as follows: 94°C for 2 min; 30 cycles of 94°C for 30 sec, 55°C for 30 sec, 72°C for 1:30; and then 72°C for 5 min. The entire volume of this second reaction was resolved on an agarose gel, and the largest distinct band was gel-purified and sent for Sanger sequencing using the primer pSAM-EC kan us3.

### Fluctuation assays

Bacteria were grown overnight in modified M9 media and then sub-cultured 1:100 into several 1 mL cultures. These were grown for either 4 h aerobically in loose-capped tubes or for 24 h microaerobically in 12-well plates. A few cultures were serially diluted and plated onto LB agar to quantify the total number of cells. The entirety of each remaining culture was then spread onto LB plates containing 20 μg/mL nalidixic acid. The following day, colonies were counted to quantify total bacterial titers as well as the numbers of nalidixic acid resistant cells. Results were analyzed by the online FALCOR calculator for fluctuation assays to determine mutation rates [50].

### RT-PCR

To probe for *rqlHI* mRNA, WT F11 was grown aerobically for 4 h in loose-capped tubes in modified M9 media after being diluted 1:100 from an overnight culture. RNA was extracted from cells using a Norgen Total RNA Purification Kit, then treated with DNase for 1.5 h, precipitated, and resuspended in water. To create cDNA, 0.5 μg of RNA was used with the Superscript III First-Strand Synthesis Kit (Life Technologies). Control reactions lacking reverse transcriptase were run concurrently. The cDNA and control reactions were then used as template in PCR reactions using two primer sets that spanned the *rqlH* and *rqlI* gene junction, or with one primer set internal to *rqlI* (S3 Table). For RT-qPCR, RNA was extracted from bacteria grow under aerobic or microaerobic conditions and cDNA produced as described above. A LightCycler 480 instrument was used to run the qPCR reactions using primers specific to *frdA*, *rqlH*, or *rqlI*, or primers that span *rqlH* and *rqlI* (S3 Table). Results were normalized to 16S rRNA levels.

### Co-immunoprecipitations and western blots

For analysis of protein complexes, a control MG1655 strain carrying the empty vectors pRR48 and pBAD33, or recombinant MG1655 strains expressing FLAG-tagged RqlI and HA-tagged RqlH or the RqlH (1–509) truncation mutant were diluted 1:100 from overnight cultures into 20 mL minimal M9 media with ampicillin and chloramphenicol. After 4 h of growth at 37°C, IPTG (1 mM) and arabinose (0.05%) were added to induce expression of RqlI and RqlH, respectively. Cultures were grown for 1 h more and bacteria were then pelleted by centrifugation and frozen. After thawing, bacterial pellets were resuspended in 500 μL B-PER Bacterial Protein Extraction Reagent (Life Technologies) containing 1X cOmplete protease inhibitor cocktail (Roche), 1 mM PMSF, and 1 μL/mL Lysonase (Merck Millipore), and then incubated at room temperature for 10 min. A small aliquot of each lysate was set aside for use as input controls for the immunoprecipitations. Lysates were incubated with Dynabeads Protein G (Life Technologies) pre-loaded with mouse anti-HA (Santa Cruz; SC-7392) or rabbit anti-FLAG (Sigma Aldrich; F7425) antibodies. After three washes in PBS, bead-protein complexes were resuspended in 30 μL sample buffer (4% sodium dodecyl sulfate [SDS], 20% glycerol, 0.02% bromophenol blue, 10% 2-mercaptoethanol, 0.125 M Tris-HCl [pH 6.8]) and heated for 5 min at 95°C in preparation for SDS–polyacrylamide gel electrophoresis (PAGE). Input controls (5% of initial lysates) for the co-immunoprecipitation experiments were similarly prepared for SDS-PAGE. Following electrophoresis, proteins were transferred to Immobilon-FL PVDF membranes (Millipore) that were then probed using antibodies specific for the FLAG or HA epitope tags or for LacI (AbCam; ab33832). Blots were developed using HRP-conjugated secondary antibodies with the BM Chemiluminescence Blotting Substrate (Roche) and imaged using a BioRad ChemiDoc MP system.

To analyze expression of the epitope-tagged RqlH and RqlI proteins and their variants, or to confirm GFP expression levels by the reporter strain MG1655*attTn7*::P_*sulA*_-GFP, bacterial cells were lysed and protein concentrations were determined as described above. Proteins from each sample were resolved by SDS-PAGE, transferred to PVDF membranes, and probed using anti-FLAG, anti-HA, or anti-GFP (Santa Cruz; SC-9996) antibodies. Subsequently, blots were stripped and probed using anti-*E*. *coli* antibody (BioDesign) to ensure equal protein loading.

### Distribution of RqlH and RqlI proteins

To find homologs of RqlH and RqlI among other bacteria, the F11 sequence of each protein was used as a query for an online blastp search using the non-redundant protein sequences database. Additionally, tblastn searches were performed against the non-redundant nucleotide and whole genome shotgun databases in order to find unannotated orthologs that would not be present in the non-redundant protein database. All BLAST searches were done with an expect threshold cutoff of 1e-06, and custom Python scripts utilizing the Biopython module [51] were used to parse the results. The hits were reciprocally blasted against the F11 proteome, and those that brought back the original query protein (i.e. RqlH or RqlI) as the top hit, aligned with at least 75% coverage, and had a percent positive score of at least 50, were defined as homologs. The species that carried homologs of only RqlH, only RqlI, or both were enumerated. To examine synteny of the *rqlH* and *rqlI* genes within bacteria that carried both genes, only hits originating from the blastp search were considered. Protein GenBank records were accessed via EFetch with Biopython, and parsed to obtain the accession numbers of the nucleotide sequences containing each *rqlH* or *rqlI* ortholog and its location within its respective genome. The gene locations were then compared to determine orientation and proximity of *rqlH* and *rqlI* to each other.

### Persister assays

Persister cell assays were performed as described [40]. Briefly, bacteria were diluted 1:100 from overnight cultures into 5 mL of LB broth and then grown with shaking (225 rpm) at 37°C for 2 h. At this point, an aliquot of each culture was taken for CFU enumeration prior to addition of ampicillin (100 μg/mL) or ciprofloxacin (10 μg/mL). After a further 5 h incubation, 1 mL of each culture was pelleted, washed once with LB broth, and surviving bacteria were determined by plating serial dilutions. Percent survival, reflecting the numbers of persister cells in each sample, was calculated by dividing the number of viable bacteria present after antibiotic treatment by the number present prior to antibiotic addition.

### Statistics

Results from *in vivo* competition assays were analyzed by one sample T tests. Results from noncompetitive assays in mice were analyzed by Mann-Whitney two-tailed *t* tests. Results from *in vitro* assays were analyzed by unpaired Student’s *t* tests. Prism 5.01 (GraphPad Software, Inc.) was used for all statistical tests, including Student’s *t* tests. P values of less than 0.05 were defined as significant.

## Supporting Information

S1 Table Strains used in this study. Includes a list of the strains used in this study and primers used to create and verify them, if applicable.
(PDF)

S2 Table Plasmids used in this study. Includes a list of the plasmids used in this study. If a plasmid was made for this study, the primers and method used to create it is included.
(PDF)

S3 Table Other primer sets used in this study. Includes primer pairs used for RT-PCR, RT-qPCR, and arbitrary PCR.
(PDF)

S1 Dataset Analysis of RqlH and RqlI distribution and synteny. Contains a list of RqlH and RqlI orthologous proteins and the genomic locations of their respective genes. Quantification of the various genera that contain RqlH and RqlI orthologs, as well as a list of all of the genera included in this analysis, are also presented.
(XLSX)

S1 Fig Activation of the SOS response in WT F11 and F11Δ*rqlI* under varying oxygen conditions. F11 (WT) or F11Δ*rqlI* (Δ) were grown in modified M9 media aerobically for 4 h or for 24 h under microaerobic or anaerobic conditions. Upper western blot shows levels of GFP expressed from strains carrying the SOS activation reporter construct P_*sulA*_-GFP (“GFP”; pJLJ3) or the empty vector control containing a promoterless GFP (“EV”; pJLJ1). Below is the same blot probed with anti-*E*. *coli* antibody, presented as a loading control.
(TIF)

S2 Fig Alignment of the RqlH proteins of F11 and *Mycobacterium smegmatis*. F11 RqlH (EcF11_3932) and M. smegmatis RqlH (MSMEG_5935) were aligned by EMBOSS Needle using the default settings. The protein alignment demonstrates that these two proteins have 41.2% identity and 55.7% similarity. The shading indicates various domains as delineated by Ordonez et al [1] and as indicated in the legend at right.
(TIF)

S3 Fig Overexpression of RqlH reduces bacterial growth, especially in the absence of RqlI. Wild type (WT), Δ*rqlH*, Δ*rqlI*, and Δ*rqlHI* cells carried either a control plasmid (pBAD33) or an arabinose-inducible rqlH expression plasmid (pCWR21). The cells were grown overnight in M9 with chloramphenicol to maintain the plasmids, and then were subcultured 1:100 into fresh M9 with chloramphenicol. The bacteria were then grown either aerobically for 4 h, or microaerobically for 6 h, at which time the cultures were titered to determine bacterial concentration. Arabinose (0.05%) was present in all cultures, added either at the time of subculturing (microaerobic), or after 2 h of growth (aerobic). Graphs show mean values ± SEM from three experiments performed in triplicate. *, P ≤ 0.05; ****, P ≤ 0.0001 as determined by Student’s *t* test.
(TIF)

S4 Fig Expression of WT and mutant RqlH proteins. MG1655*attTn7*::P_*sulA*_-GFP strains carrying the indicated RqlH expression plasmids or the empty vector pBAD33 were grown aerobically for 4 h in modified M9 media and then processed for western blot analysis. Upper blot was probed with anti-HA antibody to detect the epitope-tagged RqlH variants. *, non-specific background band present in all lanes. At the bottom is the same blot probed with anti-*E*. *coli* antibody, presented as a loading control.
(TIF)

S5 Fig The RqlH PRTase domain is responsible for loss of cell viability in the absence of RqlI. MG1655 strains carrying the empty vector pRR48 or the RqlI expression plasmid pCWR16 were transformed with pBAD33-based constructs encoding full-length RqlH or various RqlH variants, as indicated. See Fig 5 for a schematic of the WT and mutant RqlH proteins that are expressed. Strains were diluted from overnight cultures and grown aerobically in modified M9 media. After 6 h, the (A) density (OD_600_) and (B) number of viable bacteria (CFU/mL) were determined and normalized to the control strain (EV) carrying the empty vectors pRR48 and pBAD33. Graphs show mean values ± SEM from three experiments performed in triplicate. #, P<0.05 versus EV controls, as determined by Student’s *t* test. (C) Following the 6 h growth period, each recombinant strain was diluted 1:100 into fresh modified M9 media and aerobic growth in 100-well honeycomb plates was assessed using a Bioscreen C instrument. The time that it took each culture to reach an OD_600_ of 0.4 is graphed versus the log_10_-transformed data from (B). These assays were carried out in media with added IPTG and arabinose to induce expression of the recombinant RqlI and RqlH proteins.
(TIF)

S6 Fig The RqlH PRTase domain is necessary to induce filamentation and the SOS response in the absence of RqlI. MG1655 *attTn7*::P_*sulA*_-GFP carrying the empty vector pRR48 or the RqlI expression plasmid pCWR16 along with the indicated WT and mutant RqlH expression constructs were grown aerobically for 6 h in modified M9 media. IPTG and arabinose were included to induce expression of the recombinant RqlI and RqlH proteins, respectively. See Fig 5 for a schematic of the RqlH proteins that are expressed. (A) Bacterial cells were imaged and measured using ImageJ. Boxes indicate median and interquartile ranges with whiskers extending to the 1st and 99th percentiles. (B) Western blots indicate levels of GFP expression downstream of the *sulA* promoter in the recombinant strains ± RqlI, as indicated. As a loading control, blots were stripped and re-probed using anti-*E*. *coli* antibody.
(TIF)

S7 Fig Putative active site residues within the PRTase domain of RqlH. (A) PurF, Hpt, and the PRTase domain of RqlH were aligned using T-Coffee [1]. Regions corresponding to the active sites of the PurF and Hpt PRTases are shown with residues known to interact with a nucleotide highlighted in red [2,3]. Residues within RqlH that are predicted to interact with a nucleotide are highlighted in green. (B) A portion of the crystal structure of PurF showing the nucleotide-binding pocket. Adenosine monophosphate (AMP) is highlighted in green, while residues that interact with AMP are red. (C) The predicted structure of the RqlH PRTase domain generated by Phyre2 [4] was overlaid with the crystal structure of PurF. The putative nucleotide-binding pocket of RqlH is shown, in the same orientation as PurF AMP-binding pocket depicted in (B). Residues within RqlH that are predicted to be important for PRTase activity are highlighted in green (C).
(TIF)

S8 Fig Mutation of RqlH does not result in the development of anucleate bacteria. MG1655 *attTn7*::P_*sulA*_-GFP carrying pRR48 and either pBAD33 (empty vector, EV) or pCWR23 (for expression of the RqlH K49A mutant protein) were grown aerobically for 4 h in the presence of arabinose for the induction of RqlH expression. Bacterial DNA was stained with Hoechst dye, and samples were imaged by bright field and fluorescence microscopy. Representative images are shown. In the merged images, the DNA signal is false-colored yellow.
(TIF)

S9 Fig Expression of recombinant full-length and truncated RqlI variants. F11Δ*rqlI* was transformed with the empty vector pRR48 or with plasmids encoding FLAG-tagged full-length RqlI (pCWR16) or different RqlI truncation mutants, as indicated. See Fig 6 for a schematic of the RqlI variants examined. Strains were grown microaerobically for 24 h in modified M9 media. Ampicillin and IPTG were included for plasmid maintenance and for induction of RqlI expression, respectively. Upper blot shows levels of RqlI and its variants, detected using anti-FLAG antibody. *, non-specific background band. Below, as a loading control, is the same blot probed using anti-*E*. *coli* antibody.
(TIF)

S10 Fig The putative HTH domain of RqlI is dispensable, while the N-terminus is required for inhibiting RqlH toxicity. (A) WT F11 or F11Δ*rqlI* carrying empty vector (EV, pRR48) or constructs for expression of full-length and RqlI truncation mutants were grown microaerobically in modified M9 media for 24 h before titering. See Fig 6 for a schematic of the RqlI variants that were tested. Graph shows mean titers ± SEM from three experiments performed in triplicate. *, P<0.05 versus F11Δ*rqlI* control, as determined by Student’s *t* test. (B) Cultures of MG1655 expressing different RqlI variants with or without RqlH, as indicated, were grown aerobically for 6 h and then titered. Bars indicate mean titers ± SEM from three experiments performed in triplicate. *, P<0.05 versus MG1655 with empty vector (EV) control plasmids, as determined by Student’s *t* test. In these experiments, arabinose and IPTG were added to the media to induce expression of the RqlH and the RqlI variants, respectively.
(TIF)

S11 Fig RqlI is required for gut colonization by the ExPEC isolates CFT073 and 536. Adult female Balb/c mice were inoculated via oral gavage with 1x10^9^ bacteria comprised of a 1:1 mix of (A) WT CFT073 plus CFT073Δ*rqlI* or (B) WT 536 plus 536 Δ*rqlI*. Graphs show competitive indices (CI), as calculated by dilution plating of fecal homogenates on selective media at the indicated time points. Bars represent median values; n = 8 to 10 mice.
(TIF)

S12 Fig Persister cell development by WT F11 and F11Δ*rqlI*. Graph shows the percentage of surviving bacteria present 5 h after addition of ampicillin (100 mg/mL) or ciprofloxacin (10 mg/mL) to cultures in log-phase growth. Percent survival was calculated by titering cultures before and after antibiotic treatments. Data represent mean results ± SD from three independent experiments. *, P<0.05 as determined by Student’s *t* test.
(TIF)
