# Supplementary material for: The Extraintestinal Pathogenic Escherichia coli Factor RqlI Constrains the Genotoxic Effects of the RecQ-Like Helicase RqlH
Source: PLoS Pathog. 2015 Dec 4;11(12):e1005317. doi: 10.1371/journal.ppat.1005317 (PMC4670107; doi:10.1371/journal.ppat.1005317)
Supplement: S1 Table — Includes a list of the strains used in this study and primers used to create and verify them, if applicable. (PDF) [file ppat.1005317.s001.pdf]

Table S1 - Strains used in this study

Strains	Description	Reference	Primers
536	Pyelonephritis isolate	1	
536 Δ*rqlI::kan*	536 strain in which *rqlI* (*ECP_3529*) has been replaced with a kanamycin resistance cassette	This Study	KO F: TTTATCCTCTTGCCCTTGCTTCTACCTCGGTAAAAAATTG TGTGTAGGCTGGAGCTGCTTCG<br>KO R: AAACAGGTGTTGCACACAACCAGACGCGGCACCTGTTTTA CATATGAATATCCTCCTTAG<br>Confirmation F: TTATCCTCTTGCCCTTGCTT<br>Confirmation R: CGAATGTGGATGAAGGGGTA
536::clm	536 with chromosomal insertion of chloramphenicol resistance cassette at the intergenic region between *ECP_2512* and *ECP_2511*	This Study	KO F: TCTGGCGTAGCCTGGGAGTTATTGCCGGATGCGATGCTGG TGTGTAGGCTGGAGCTGCTTCG<br>KO R: TCACGTAAAAAAACGTCTAATCCGTAGACCGGATAAGAGG CATATGAATATCCTCCTTAG<br>Confirmation F:<br>Confirmation R:
CFT073	Pyelonephritis isolate	2	
CFT073 Δ*rqlI::kan*	CFT073 strain in which *rqlI* (*c4222*) has been replaced with a kanamycin resistance cassette	This Study	KO F: TTTATCCTCTTGCCCTTGCTTCTACCTCGGTAAAAAATTG TGTGTAGGCTGGAGCTGCTTCG<br>KO R: AAACAGGTGTTGCACACAACCAGACGCGGCACCTGTTTTA CATATGAATATCCTCCTTAG<br>Confirmation F: TTATCCTCTTGCCCTTGCTT<br>Confirmation R: CGAATGTGGATGAAGGGGTA
CFT073::kan	CFT073 with chromosomal insertion of kanamycin resistance cassette at the intergenic region between genes *c3028* and *c3029*	This Study	KO F: TCTGGCGTAGCCTGGGAGTTATTGCCGGATGCGATGCTGG TGTGTAGGCTGGAGCTGCTTCG<br>KO R: TCACGTAAAAAAACGTCTAATCCGTAGACCGGATAAGAGG CATATGAATATCCTCCTTAG<br>Confirmation F: GGATGGACGAAGGTACTGGA<br>Confirmation R: GGTGAGACACTGACCACACG
F11	Cystitis isolate	3	
F11::clm	F11 with chromosomal insertion of chloramphenicol resistance cassette at the intergenic region between genes *EcF11_2526* and *xseA*	This study	KO F: TCTGGCGTAGCCTGGGAGTTATTGCCGGATGCGATGCTGG TGTGTAGGCTGGAGCTGCTTCG<br>KO R: TCACGTAAAAAAACGTCTAATCCGTAGACCGGATAAGAGG CATATGAATATCCTCCTTAG<br>Confirmation F: GGATGGACGAAGGTACTGGA<br>Confirmation R: GGTGAGACACTGACCACACG
F11 Δ*rqlI::kan*	F11 strain in which *rqlI* (*EcF11_3933*) has been replaced with a kanamycin resistance cassette	4	
F11 Δ*rqlI*	F11 strain in which *rqlI* has been fully deleted by flipping out the antibiotic resistance cassette	This Study	
F11 Δ*rqlH::kan*	F11 strain in which *rqlH* (*EcF11_3932*) has been replaced with a kanamycin resistance cassette	This Study	KO F: TCTAATCACTGCGTTAACGGACGAGGGAGAAGATGATGTGTGTAGGCTGGAGCTGCTTCG<br>KO R: GCATTGGCTGAAAGATTCATCAATTTTTTACCGAGGTAGACATATGAATATCCTCCTTAG<br>Confirmation F: ATCCGTTCTAATCACTGCGT<br>Confirmation R: CTTGTGCATTGGCTGAAAGA
F11 Δ*rqlH*	F11 strain in which *rqlH* has been fully deleted	This Study	
F11 Δ*rqlHI::clm*	F11 strain in which the *rqlHI* operon has been replaced with a chloramphenicol resistance cassette	This Study	KO F: TCTAATCACTGCGTTAACGGACGAGGGAGAAGATGATGTGTGTAGGCTGGAGCTGCTTCG<br>KO R: AAACAGGTGTTGCACACAACCAGACGCGGCACCTGTTTTA CATATGAATATCCTCCTTAG<br>Confirmation F: ATCCGTTCTAATCACTGCGT<br>Confirmation R: CGAATGTGGATGAAGGGGTA
F11 Δ*lacZY::clm*	F11 strain in which *lacZY* has been replaced with a chloramphenicol cassette	This Study	KO F: ATGACTATGATTACGGATTCTCTGGCCGTCGTATTACATGTGTAGGCTGGAGCTGCTTCG<br>KO R: TTAAGCGACTTCATTCACCTGACGACGCAGTAGAGAAAGCCATATGAATATCCTCCTTAG<br>Confirmation F: CTAAGGAGGATATTCATATG<br>Confirmation R: CCTACCGTGGCAAACATTTC
F11 Δ*rqlI* Δ*lacZY::clm*	F11 strain in which *rqlI* has been fully deleted and *lacZY* has been replaced with a chloramphenicol resistance cassette	This Study	
MG1655::*tetA-sacB*	MG1655 strain in which the *tetA-sacB* cassette from T-SACK has been inserted at the *attTn7* site	This Study	Upstream F: GGCACCAAAATCAACGGTAT<br>Upstream R: GATAGAGTGTCAACAAAAATTAGGAGCCAACTCCATGTGACAAACC<br>tetA-sacB F: TCCTAATTTTTGTTGACACTCTATC<br>tetA-sacB R: TTAATCAAAGGGAAAACTGTCCATATGC<br>Downstream F: GCATATGGACAGTTTTCCCTTTGATTAACTTACGCAGGGCATCCATTT<br>Downstream R: TCAGGACAAACGCATTGAAG
MG1655::P_{sulA}-*GFP*	MG1655 strain in which the *tetA-sacB* cassette has been replaced by the P_{sulA}-*GFP* reporter construct at the *attTn7* site	This Study	Upstream F: GGCACCAAAATCAACGGTAT<br>Upstream R: CTATTGGATCCTCCGGCGTTGCCAACTCCATGTGACAAACC<br>PsulA-GFP F: AACGCCGGAGGATCCAATAG<br>PsulA-GFP R: CGGCCGACTAGTAGGCCTATTA<br>Downstream F: TAATAGGCCTACTAGTCGGCCGCTTACGCAGGGCATCCATTT<br>Downstream R: TCAGGACAAACGCATTGAAG

(1) Hacker J, Hughes C. Genetics of *Escherichia coli* hemolysin. Curr Top Microbiol Immunol. 1985;118:139-62.

(2) Mobley HL, Green DM, Trifillis AL, Johnson DE, Chippendale GR, et al. Defining genomic islands and uropathogen-specific genes in uropathogenic *Escherichia coli*. J Bacteriol. 2007 May;189(9):3532-46.

(3) Stapleton A, Moseley S, Stamm WE. Urovirulence determinants in *Escherichia coli* isolates causing first-episode and recurrent cystitis in women. J Infect Dis. 1991 Apr;163(4):773-9.

(4) Wiles TJ, Norton JP, Russell CW, Dalley BK, Fischer KF, Mulvey MA. Combining quantitative genetic footprinting and trait enrichment analysis to identify fitness determinants of a bacterial pathogen. PLoS Genet. 2013;9(8):e1003716.
