# Supplementary material for: The Extraintestinal Pathogenic Escherichia coli Factor RqlI Constrains the Genotoxic Effects of the RecQ-Like Helicase RqlH
Source: PLoS Pathog. 2015 Dec 4;11(12):e1005317. doi: 10.1371/journal.ppat.1005317 (PMC4670107; doi:10.1371/journal.ppat.1005317)
Supplement: S2 Table — Includes a list of the plasmids used in this study. If a plasmid was made for this study, the primers and method used to create it is included. (PDF) [file ppat.1005317.s002.pdf]

Table S2 - Plasmids used in this study

Plasmid	Description	Reference	Primers
pBAD33	Empty vector used to create arabinose-inducible expression plasmids	1	
pRR48	Empty vector used to create IPTG-inducible expression plasmids	2	
pKM208	Carries an IPTG-inducible lambda red system used for recombineering	3	
pGEN-MCS	Empty vector that carries the hok-sok TA system for retainment of the plasmid	4	
pJLJ1	pGFP was cut with BamHI and BsiWI, and the resulting GFP (ASV) fragment was ligated into pACYC177 digested with BamHI and BanI. Used as a promoterless control for pJLJ3	This Study	
pJLJ3	The *sulA* promoter was inserted into the BamHI and Asp718 sites of pGFP. The resulting plasmid was then cut with BamHI and BsiWI, and the P_{sulA}-GFP (ASV) fragment was ligated into pACYC177 cut with BamHI and BanI	This Study	GATCCAATAGGGTTGATCTTTGTTGTCACTGGATGTACTGTACATCCATACAGTAA<br>GTACCTTACTGTATGGATGTACAGTACATCCAGTGACAACAAAGATCAACCCTATT
pCWR1	The promoterless *lacZ* gene from MG1655 was inserted between the NcoI and HindIII sites of pGEN-MCS using overlap extension PCR	This Study	GCCATTGAAAAAGTCGTGGACAATCCACCG ATGACCATGATTACGGATTC<br>CTATCCCTTATCGTCGTCATCCTTGTAGTCCCT TTATTTTTGACACCAGACCA
pCWR2	The 330 nucleotides upstream of the *sulA* start site in F11, plus the first 30 nucleotides of *sulA* were added into pCWR1 just upstream of *lacZ* via overlap extension PCR	This Study	CGTTTGCACTGTCTCTGTTATTCAGGCAATTTCCGTGTCGTCAACGGTCAGGC<br>AACGACGGCCAGTGAATCCGTAATCATGGTAGAACGATGTGCATAGCCTG
pCWR6	Amino acids 1-250 of F11 RqlI with a FLAG tag on the N-terminus were inserted into the HindIII and PstI sites of pRR48	This Study	CCCGGCTGCAGATGGACTACAAGGATGACGACGATAAGGGAATGAATCTTTCAGCCAATG<br>GGGCCAAGCTTCTATCCCGCACGTACAACCATTG
pCWR7	Amino acids 1-320 of F11 RqlI with a FLAG tag on the N-terminus were inserted into the HindIII and PstI sites of pRR48	This Study	CCCGGCTGCAGATGGACTACAAGGATGACGACGATAAGGGAATGAATCTTTCAGCCAATG<br>GGGCCAAGCTTCTATTGAGCGTTACGGAGTGCTC
pCWR8J	Amino acids 320-400 of F11 RqlI with a FLAG tag on the N-terminus were inserted into the HindIII and PstI sites of pRR48	This Study	CCCGGCTGCAGATGGACTACAAGGATGACGACGATAAGGGACAACCAGAACTGTTTTCGC<br>GGGCCAAGCTTTTACCTGAACTGATAATGA
pCWR11	Amino acids 130-320 of F11 RqlI with a FLAG tag on the N-terminus were inserted into the HindIII and PstI sites of pRR48	This Study	CCCGGCTGCAGATGGACTACAAGGATGACGACGATAAGGGAGCGGAAGGTATGGCGATTG<br>GGGCCAAGCTTCTATTGAGCGTTACGGAGTGCTC
pCWR16	Full-length F11 RqlI with a FLAG tag on the N-terminus was inserted into the HindIII and PstI sites of pRR48	This Study	CCCGGCTGCAGATGGACTACAAGGATGACGACGATAAGGGAATGAATCTTTCAGCCAATG<br>GGGCCAAGCTTTTACCTGAACTGATAATGAC
pCWR21	A C-terminally HA tagged F11 RqlH with a GGGGP linker between the HA tag and RqlH, inserted into HindIII and SacI sites of pBAD33	This Study	GCCGGGAGCTCAGGAGGACTGACTGATGGAGAAACACGGAGCTGA<br>GCCGGAAGCTTTCAAGCGTAATCTGGAACATCGTATGGGTATGGTCCTCCTCCTCCATTTTTTACCGAGGTAGAAGCAAGG
pCWR22	Amino acids 85-320 of F11 RqlI with a FLAG tag on the N-terminus were inserted into the HindIII and PstI sites of pRR48	This Study	CCCGGCTGCAGATGGACTACAAGGATGACGACGATAAGGGAGCGGTAGATAAGTGGCAAC<br>GGGCCAAGCTTCTATTGAGCGTTACGGAGTGCTC
pCWR23	A K49A mutant in RqlH. Created with site-directed mutagenesis using pCWR21 as template.	This Study	CGCACCGGCTGGGGGGCTAGTGCCGTGTACTTC<br>GAAGTACACGGCACTAGCCCCCCAGCCGGTGCG
pCWR24	A D148A mutant in RqlH. Created with site-directed mutagenesis using pCWR21 as template.	This Study	GGTTTACTGGTGGTCGCTGAGGCGCACTGCATC<br>GATGCAGTGCGCCTC AGC GACCACCAGTAAACC
pCWR25	Amino acids 1-509 of F11 RqlH with an HA tag on the C-terminus were inserted into pBAD33 at the HindIII and SacI sites. A GGGGP linker is located between RqlH and the HA tag.	This Study	GCCGGGAGCTCAGGAGGACTGACTGATGGAGAAACACGGAGCTGA<br>GCCGGAAGCTTTCAAGCGTAATCTGGAACATCGTATGGGTATGGTCCTCCTCCTCCGTCAGCGTGTTTAATAAATAAATTTGC
pCWR26	Amino acids 509-698 of F11 RqlH and an HA tag on the N-terminus were inserted into pBAD33 at the HindIII and SacI sites. A GGGGP linker is located between RqlH and the HA tag	This Study	GCCGGGAGCTCAGGAGGACTGACTGATGTACCCATACGATGTTCCAGATTACGCTGGAGGAGGAGGACCAGACCTGCCATTAAATCTCAA<br>GCCGGAAGCTTTCAATTTTTTACCGAGGTAGAAGCA
pCWR28	A DIVD663EVME mutation in RqlH. Created with site-directed mutagenesis using pCWR21 as template	This Study	GTCCGGCGTTGCTGGTTGATGAGGTCATGGAGTCTGCATGGACGC<br>GCGTCCATGCAGACTCCATGACCTCATCAACCAGCAACGCCGGAC
pCWR29	A D663A mutation in RqlH. Created with site-directed mutagenesis using pCWR21 as template	This Study	GGCGTTGCTGGTTGATGCTATCGTGGATTCTGCAT<br>ATGCAGAATCCACGATAGCATCAACCAGCAACGCC
pCWR30	A K49A and D663A mutation in RqlH. Created by adding D663A mutation to pCWR23 by site-directed mutagenesis	This Study	GGCGTTGCTGGTTGATGCTATCGTGGATTCTGCAT<br>ATGCAGAATCCACGATAGCATCAACCAGCAACGCC

(1) Guzman LM, Belin D, Carson MJ, Beckwith J. Tight regulation, modulation, and high-level expression by vectors containing the arabinose PBAD promoter. J Bacteriol. 1995 Jul;177(14):4121-30.

(2) Studdert CA, Parkinson JS. Insights into the organization and dynamics of bacterial chemoreceptor clusters through in vivo crosslinking studies. Proc Natl Acad Sci U S A. 2005 Oct 25;102(43):15623-8.

(3) Murphy KC, Campellone KG. Lambda Red-mediated recombinogenic engineering of enterohemorrhagic and enteropathogenic *E. coli*. BMC Mol Biol. 2003 Dec 13;4:11.

(4) Lane MC, Alteri CJ, Smith SN, Mobley HL. Expression of flagella is coincident with uropathogenic *Escherichia coli* ascension to the upper urinary tract. Proc Natl Acad Sci U S A. 2007 Oct 16;104(42):16669-74.
