# Supplementary material for: The Extraintestinal Pathogenic Escherichia coli Factor RqlI Constrains the Genotoxic Effects of the RecQ-Like Helicase RqlH
Source: PLoS Pathog. 2015 Dec 4;11(12):e1005317. doi: 10.1371/journal.ppat.1005317 (PMC4670107; doi:10.1371/journal.ppat.1005317)
Supplement: S3 Table — Includes primer pairs used for RT-PCR, RT-qPCR, and arbitrary PCR. (PDF) [file ppat.1005317.s003.pdf]

Primers for qPCR (Figure 4C)

Target	Primer Sequences
16S	ATGACCAGCCACACTGGAACT<br>AGTATCAGATGCAGTTCCCAG
rqlI	CTGGCCCAACAGGGGATTT<br>CTCCATCGCCCCGGTTATCG
rqlH	AACACGCTGACCTGCCATTA<br>AATCAGGAACCAGGTCGAGG
rqlHI spanning	TTACCCCTCCTTTGATGCCA<br>GCTCCAGCCTGTAAGTTTCT
frdA	AGTATACCCGATGCGTAGCC<br>TGCAGAGAGGTCTGGAACAG

Primers for semiquantitative PCR (Figure 4B)

Primer	Sequence
RqlI Reverse	CTCCATCGCCCCGGTTATCG
RqlI Forward (#1)	TCAGCCAATGCACAAGCAAC
RqlH Forward (#2)	AACACGCTGACCTGCCATTA
RqlH Forward (#3)	TTATCCTCTTGCCCTTGCTT

Primers for arbitrary PCR (Figure 3)

Primer	Sequence
ARB1A	GCCACGCGTCGACTAGTACNNNNNNNNNNACGCC
ARB1B	GCCACGCGTCGACTAGTACNNNNNNNNNNTGCGG
ARB1C	GCCACGCGTCGACTAGTACNNNNNNNNNNTCCGG
pSAM-EC kan us1	TGTCCATAAAACCGCCCAGT
ARB2	GCCACGCGTCGACTAGTAC
pSAM-EC kan us2	ATGTAAGCCCACTGCAAGCT
pSAM-EC kan us3	CCGCTTCCTCAATTGCTCGA
