# Supplementary figures and images for: The Extraintestinal Pathogenic Escherichia coli Factor RqlI Constrains the Genotoxic Effects of the RecQ-Like Helicase RqlH
Source: PLoS Pathog. 2015 Dec 4;11(12):e1005317. doi: 10.1371/journal.ppat.1005317 (PMC4670107; doi:10.1371/journal.ppat.1005317)

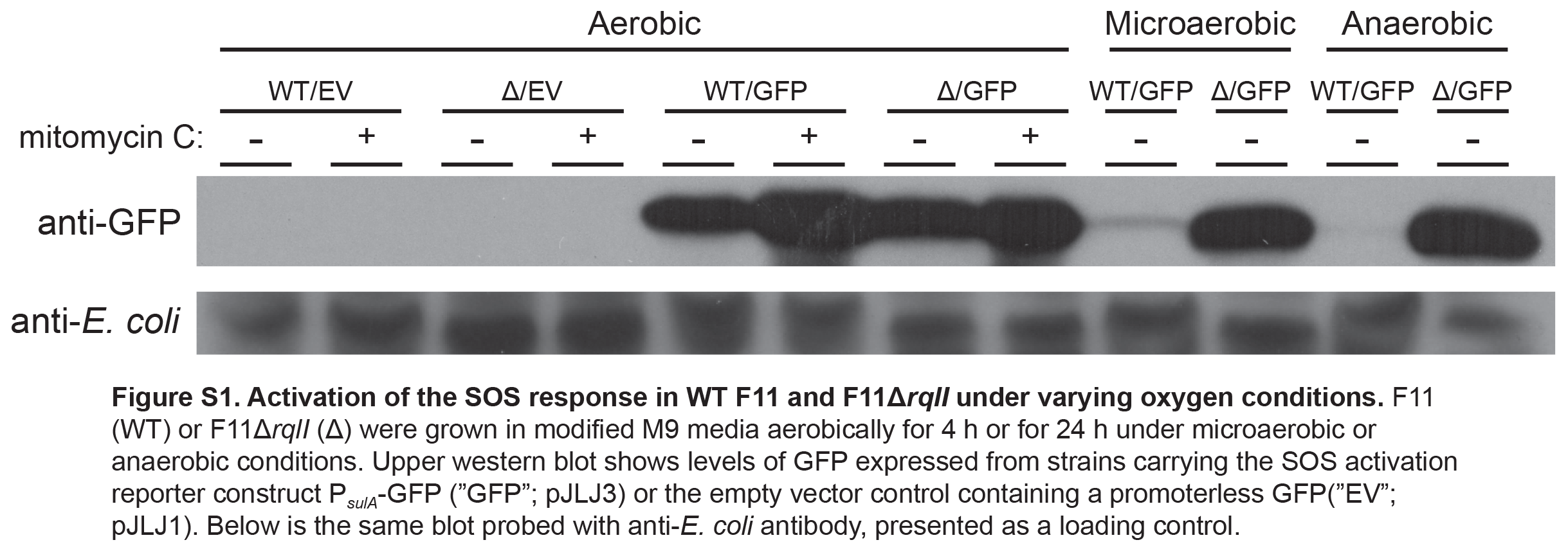

Supplement: S1 Fig — F11 (WT) or F11ΔrqlI (Δ) were grown in modified M9 media aerobically for 4 h or for 24 h under microaerobic or anaerobic conditions. Upper western blot shows levels of GFP expressed from strains carrying the SOS activation reporter construct PsulA-GFP (“GFP”; pJLJ3) or the empty vector control containing a promoterless GFP (“EV”; pJLJ1). Below is the same blot probed with anti-E. coli antibody, presented as a loading control. (TIF) [file ppat.1005317.s005.tif]

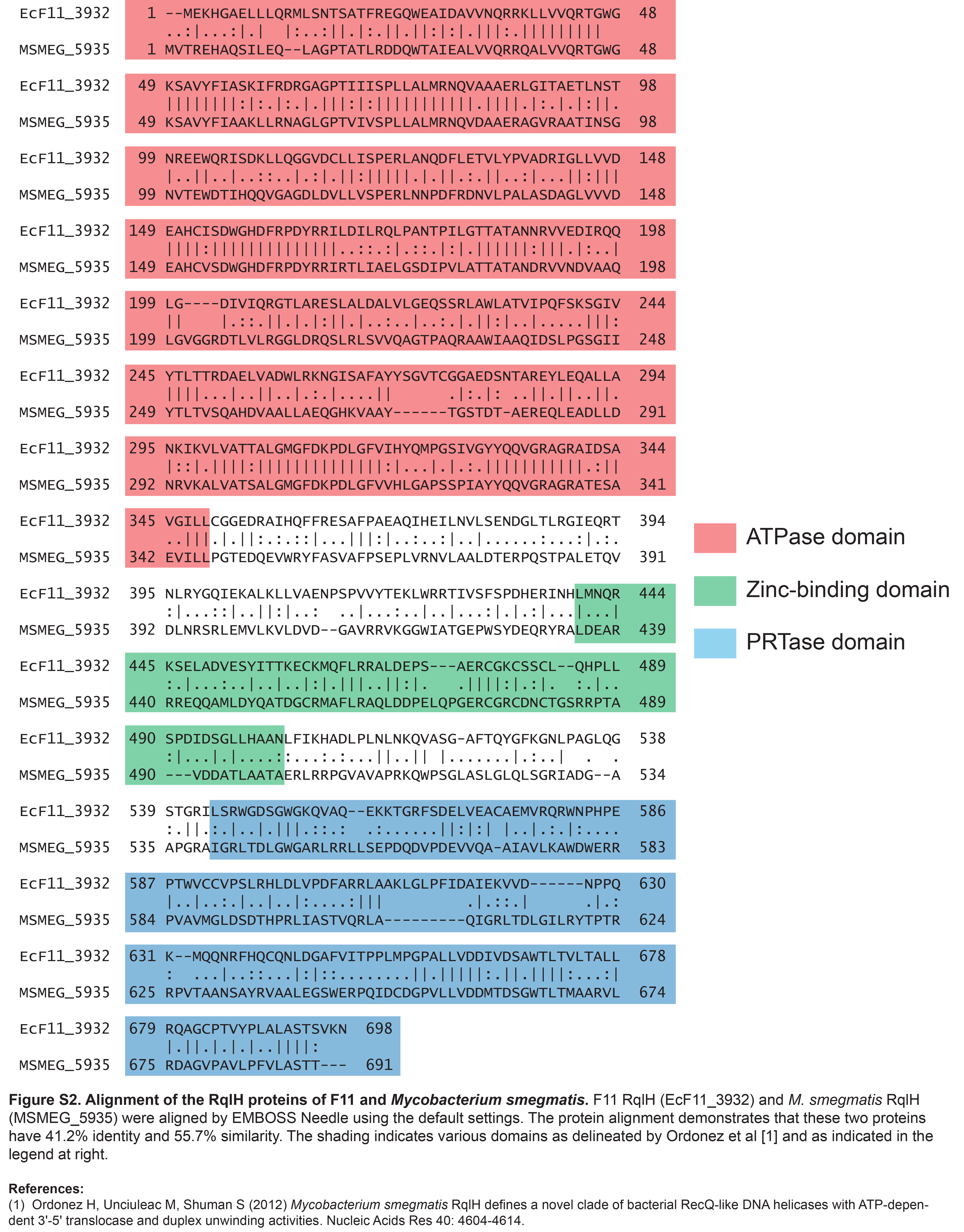

Supplement: S2 Fig — F11 RqlH (EcF11_3932) and M. smegmatis RqlH (MSMEG_5935) were aligned by EMBOSS Needle using the default settings. The protein alignment demonstrates that these two proteins have 41.2% identity and 55.7% similarity. The shading indicates various domains as delineated by Ordonez et al [1] and as indicated in the legend at right. (TIF) [file ppat.1005317.s006.tif]

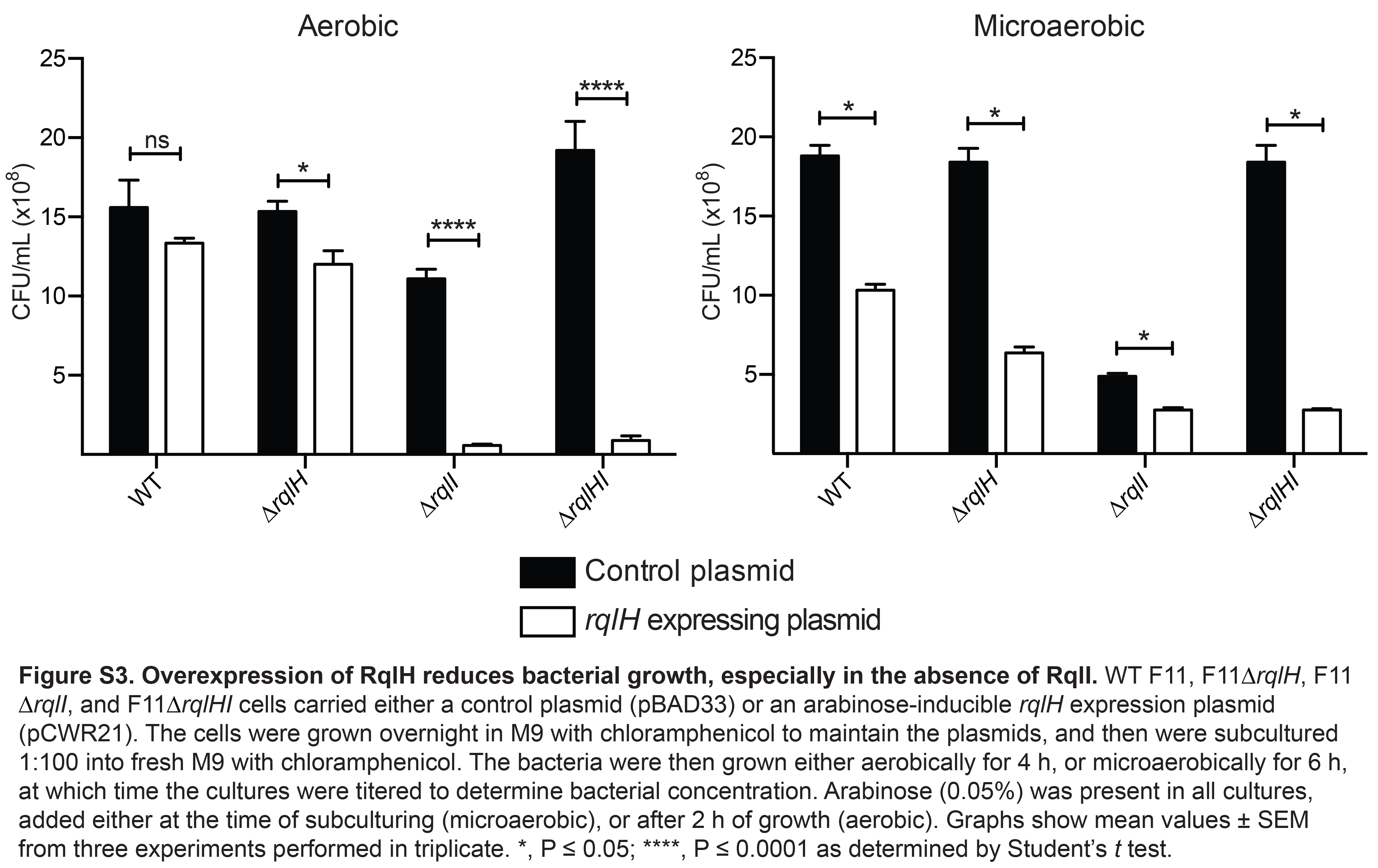

Supplement: S3 Fig — Wild type (WT), ΔrqlH, ΔrqlI, and ΔrqlHI cells carried either a control plasmid (pBAD33) or an arabinose-inducible rqlH expression plasmid (pCWR21). The cells were grown overnight in M9 with chloramphenicol to maintain the plasmids, and then were subcultured 1:100 into fresh M9 with chloramphenicol. The bacteria were then grown either aerobically for 4 h, or microaerobically for 6 h, at which time the cultures were titered to determine bacterial concentration. Arabinose (0.05%) was present in all cultures, added either at the time of subculturing (microaerobic), or after 2 h of growth (aerobic). Graphs show mean values ± SEM from three experiments performed in triplicate. *, P ≤ 0.05; ****, P ≤ 0.0001 as determined by Student’s t test. (TIF) [file ppat.1005317.s007.tif]

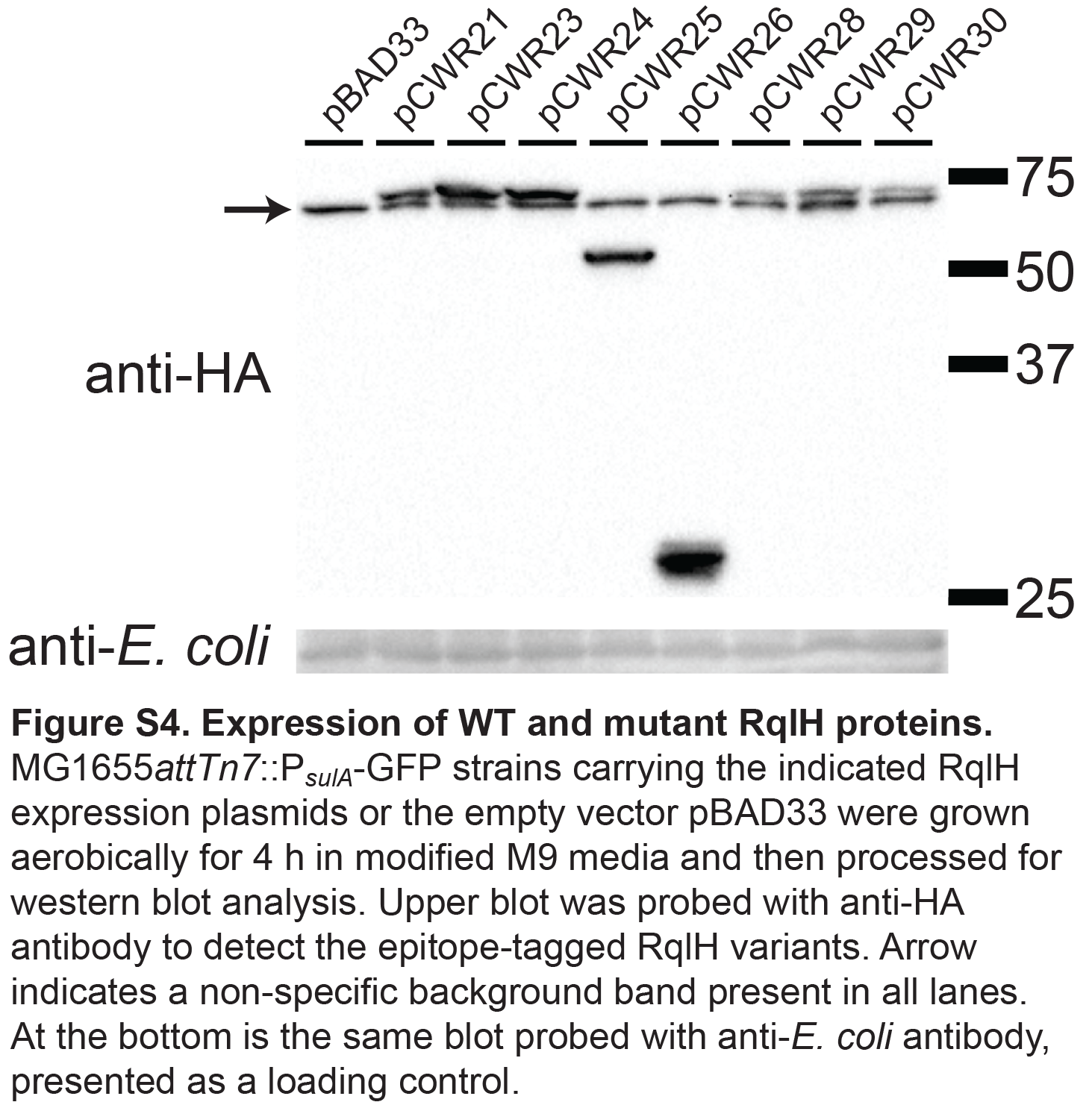

Supplement: S4 Fig — MG1655attTn7::PsulA-GFP strains carrying the indicated RqlH expression plasmids or the empty vector pBAD33 were grown aerobically for 4 h in modified M9 media and then processed for western blot analysis. Upper blot was probed with anti-HA antibody to detect the epitope-tagged RqlH variants. *, non-specific background band present in all lanes. At the bottom is the same blot probed with anti-E. coli antibody, presented as a loading control. (TIF) [file ppat.1005317.s008.tif]

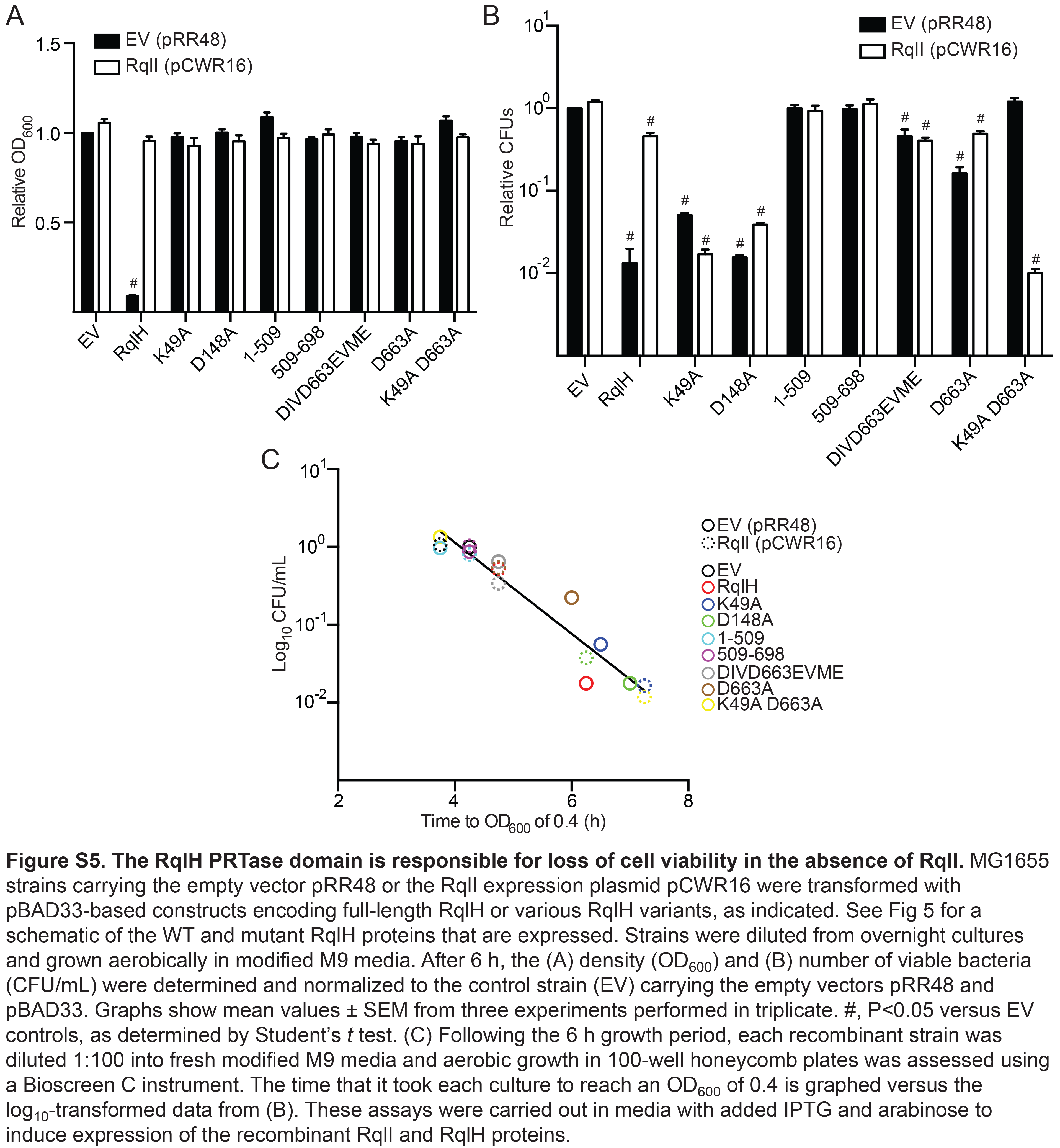

Supplement: S5 Fig — MG1655 strains carrying the empty vector pRR48 or the RqlI expression plasmid pCWR16 were transformed with pBAD33-based constructs encoding full-length RqlH or various RqlH variants, as indicated. See Fig 5 for a schematic of the WT and mutant RqlH proteins that are expressed. Strains were diluted from overnight cultures and grown aerobically in modified M9 media. After 6 h, the (A) density (OD600) and (B) number of viable bacteria (CFU/mL) were determined and normalized to the control strain (EV) carrying the empty vectors pRR48 and pBAD33. Graphs show mean values ± SEM from three experiments performed in triplicate. #, P<0.05 versus EV controls, as determined by Student’s t test. (C) Following the 6 h growth period, each recombinant strain was diluted 1:100 into fresh modified M9 media and aerobic growth in 100-well honeycomb plates was assessed using a Bioscreen C instrument. The time that it took each culture to reach an OD600 of 0.4 is graphed versus the log10-transformed data from (B). These assays were carried out in media with added IPTG and arabinose to induce expression of the recombinant RqlI and RqlH proteins. (TIF) [file ppat.1005317.s009.tif]

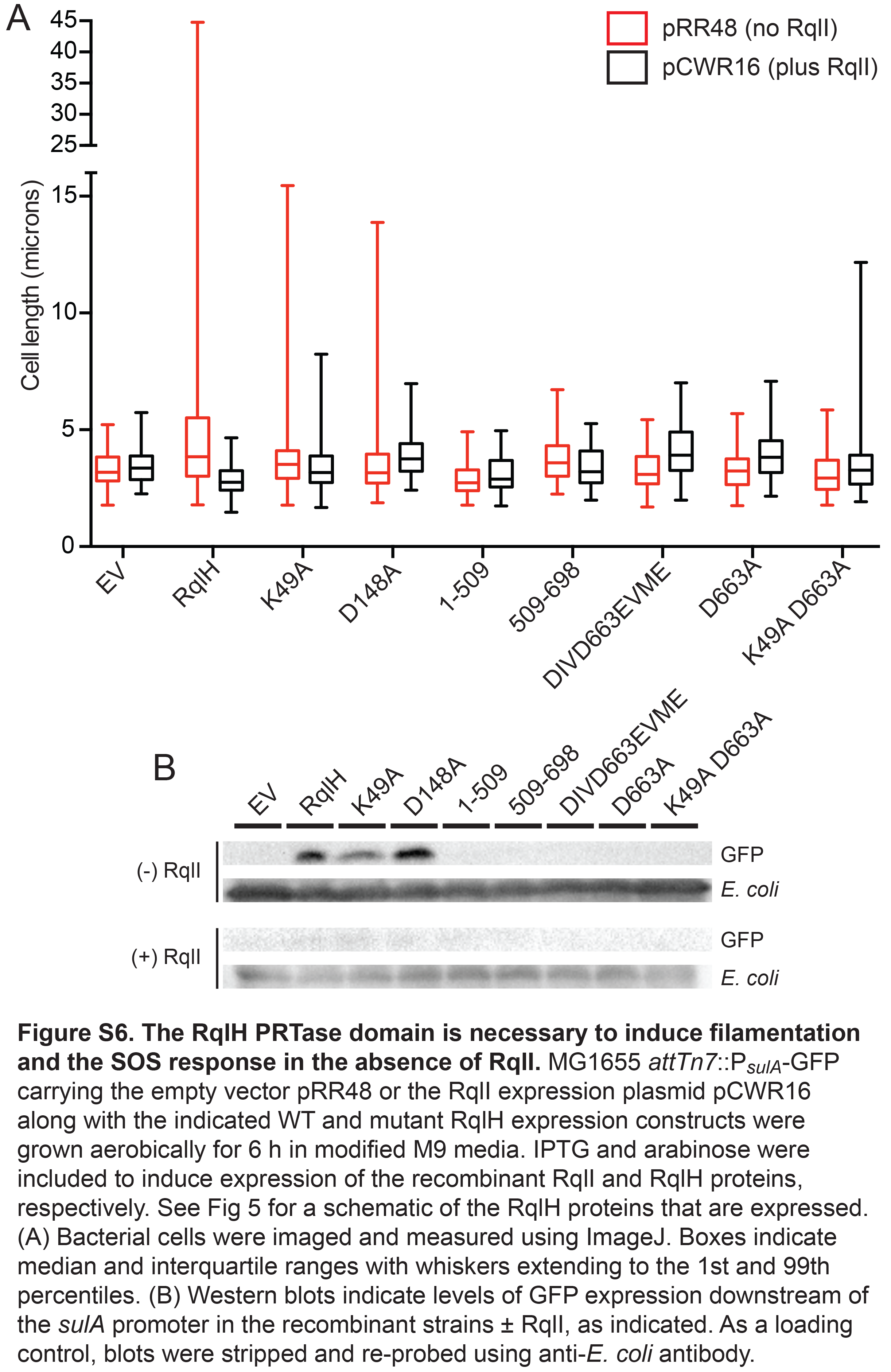

Supplement: S6 Fig — MG1655 attTn7::PsulA-GFP carrying the empty vector pRR48 or the RqlI expression plasmid pCWR16 along with the indicated WT and mutant RqlH expression constructs were grown aerobically for 6 h in modified M9 media. IPTG and arabinose were included to induce expression of the recombinant RqlI and RqlH proteins, respectively. See Fig 5 for a schematic of the RqlH proteins that are expressed. (A) Bacterial cells were imaged and measured using ImageJ. Boxes indicate median and interquartile ranges with whiskers extending to the 1st and 99th percentiles. (B) Western blots indicate levels of GFP expression downstream of the sulA promoter in the recombinant strains ± RqlI, as indicated. As a loading control, blots were stripped and re-probed using anti-E. coli antibody. (TIF) [file ppat.1005317.s010.tif]

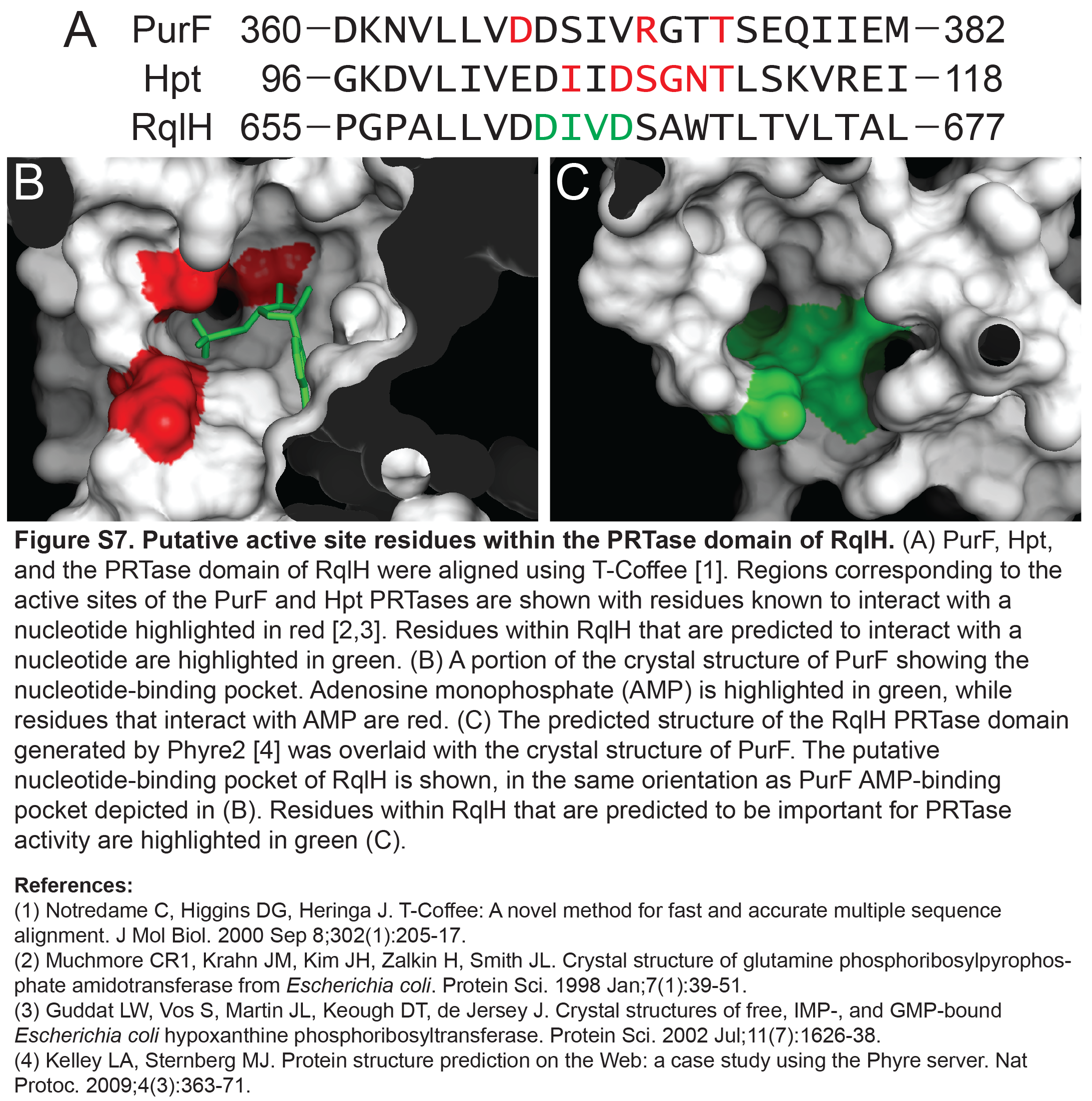

Supplement: S7 Fig — (A) PurF, Hpt, and the PRTase domain of RqlH were aligned using T-Coffee [1]. Regions corresponding to the active sites of the PurF and Hpt PRTases are shown with residues known to interact with a nucleotide highlighted in red [2,3]. Residues within RqlH that are predicted to interact with a nucleotide are highlighted in green. (B) A portion of the crystal structure of PurF showing the nucleotide-binding pocket. Adenosine monophosphate (AMP) is highlighted in green, while residues that interact with AMP are red. (C) The predicted structure of the RqlH PRTase domain generated by Phyre2 [4] was overlaid with the crystal structure of PurF. The putative nucleotide-binding pocket of RqlH is shown, in the same orientation as PurF AMP-binding pocket depicted in (B). Residues within RqlH that are predicted to be important for PRTase activity are highlighted in green (C). (TIF) [file ppat.1005317.s011.tif]

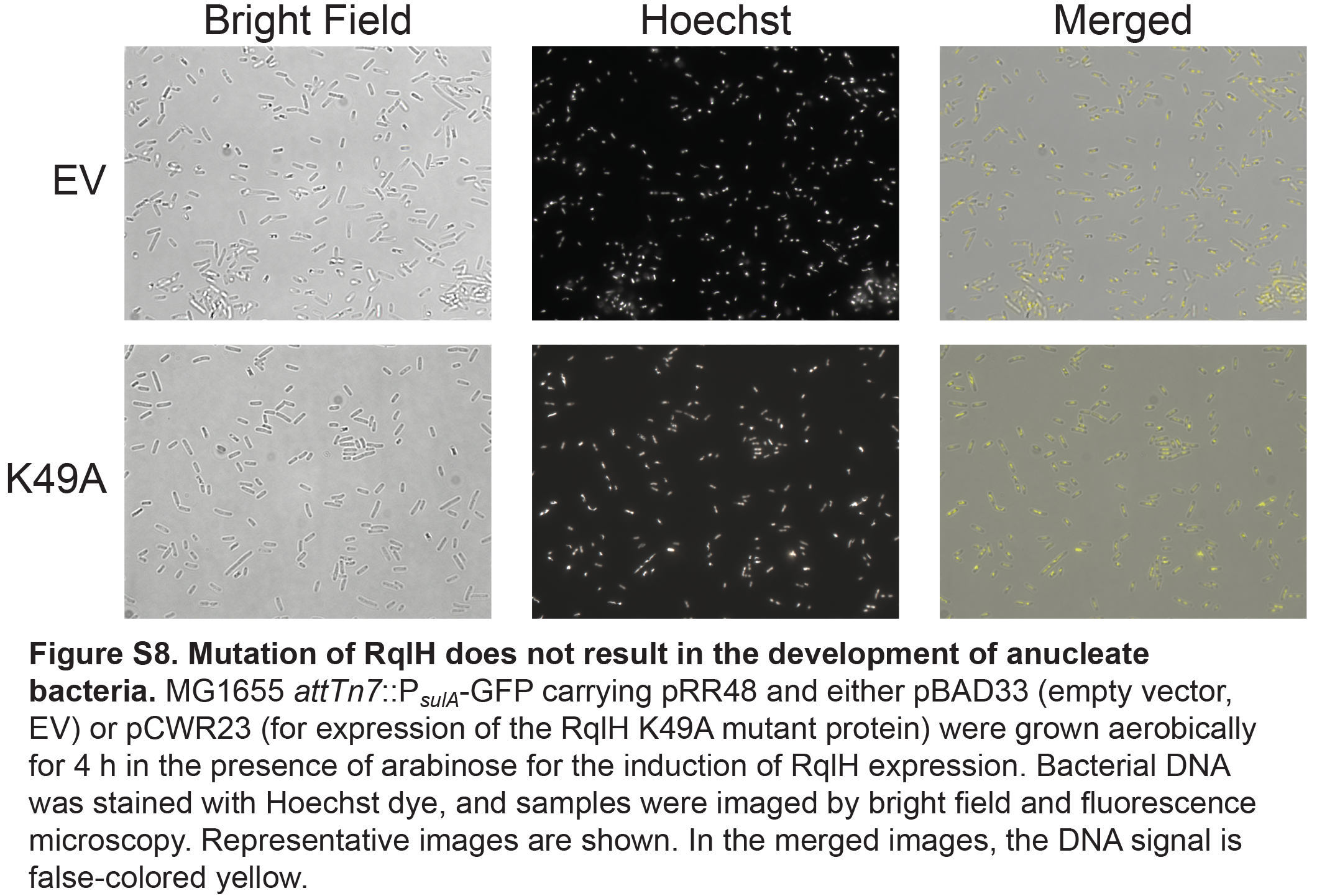

Supplement: S8 Fig — MG1655 attTn7::PsulA-GFP carrying pRR48 and either pBAD33 (empty vector, EV) or pCWR23 (for expression of the RqlH K49A mutant protein) were grown aerobically for 4 h in the presence of arabinose for the induction of RqlH expression. Bacterial DNA was stained with Hoechst dye, and samples were imaged by bright field and fluorescence microscopy. Representative images are shown. In the merged images, the DNA signal is false-colored yellow. (TIF) [file ppat.1005317.s012.tif]

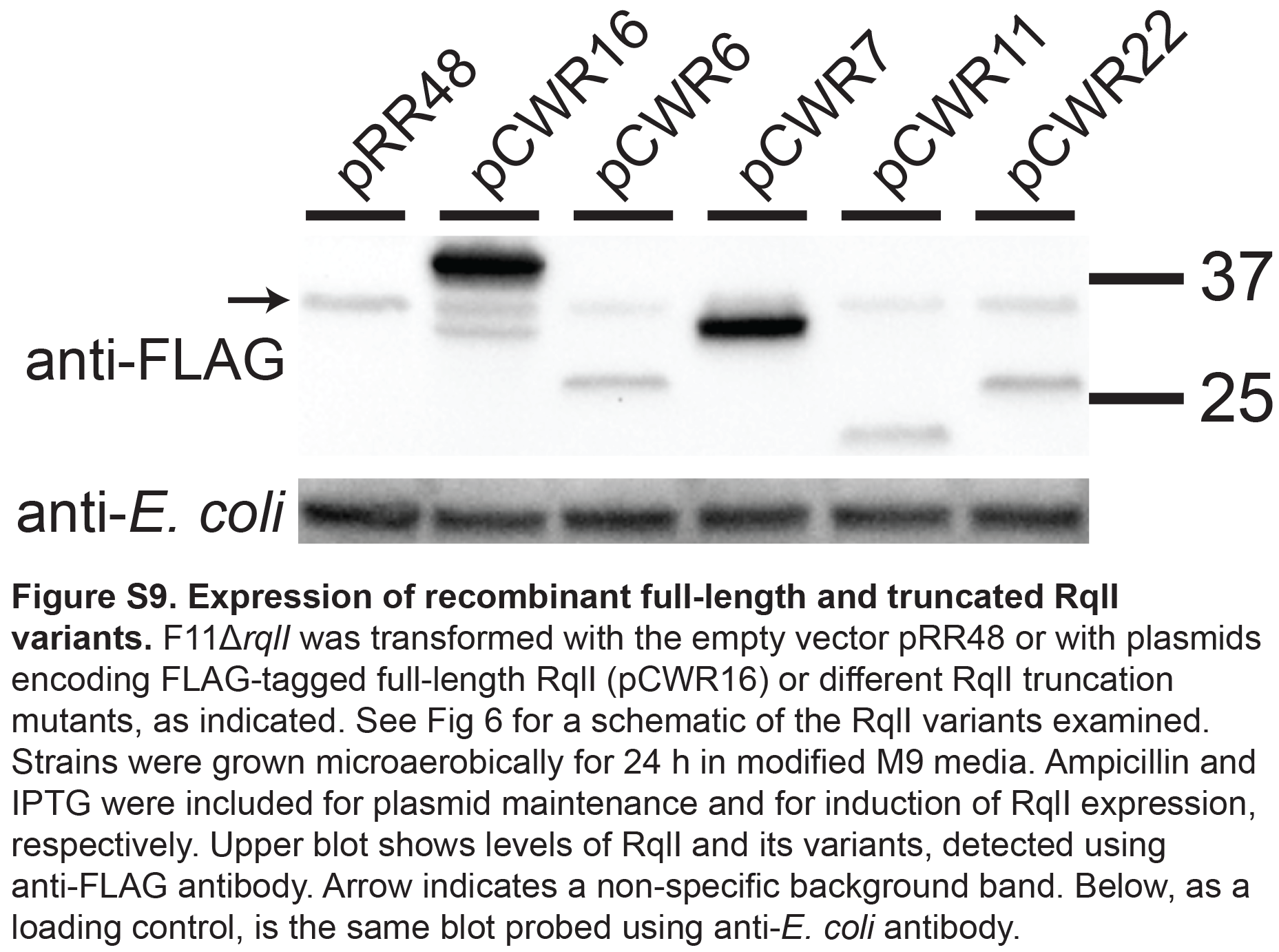

Supplement: S9 Fig — F11ΔrqlI was transformed with the empty vector pRR48 or with plasmids encoding FLAG-tagged full-length RqlI (pCWR16) or different RqlI truncation mutants, as indicated. See Fig 6 for a schematic of the RqlI variants examined. Strains were grown microaerobically for 24 h in modified M9 media. Ampicillin and IPTG were included for plasmid maintenance and for induction of RqlI expression, respectively. Upper blot shows levels of RqlI and its variants, detected using anti-FLAG antibody. *, non-specific background band. Below, as a loading control, is the same blot probed using anti-E. coli antibody. (TIF) [file ppat.1005317.s013.tif]

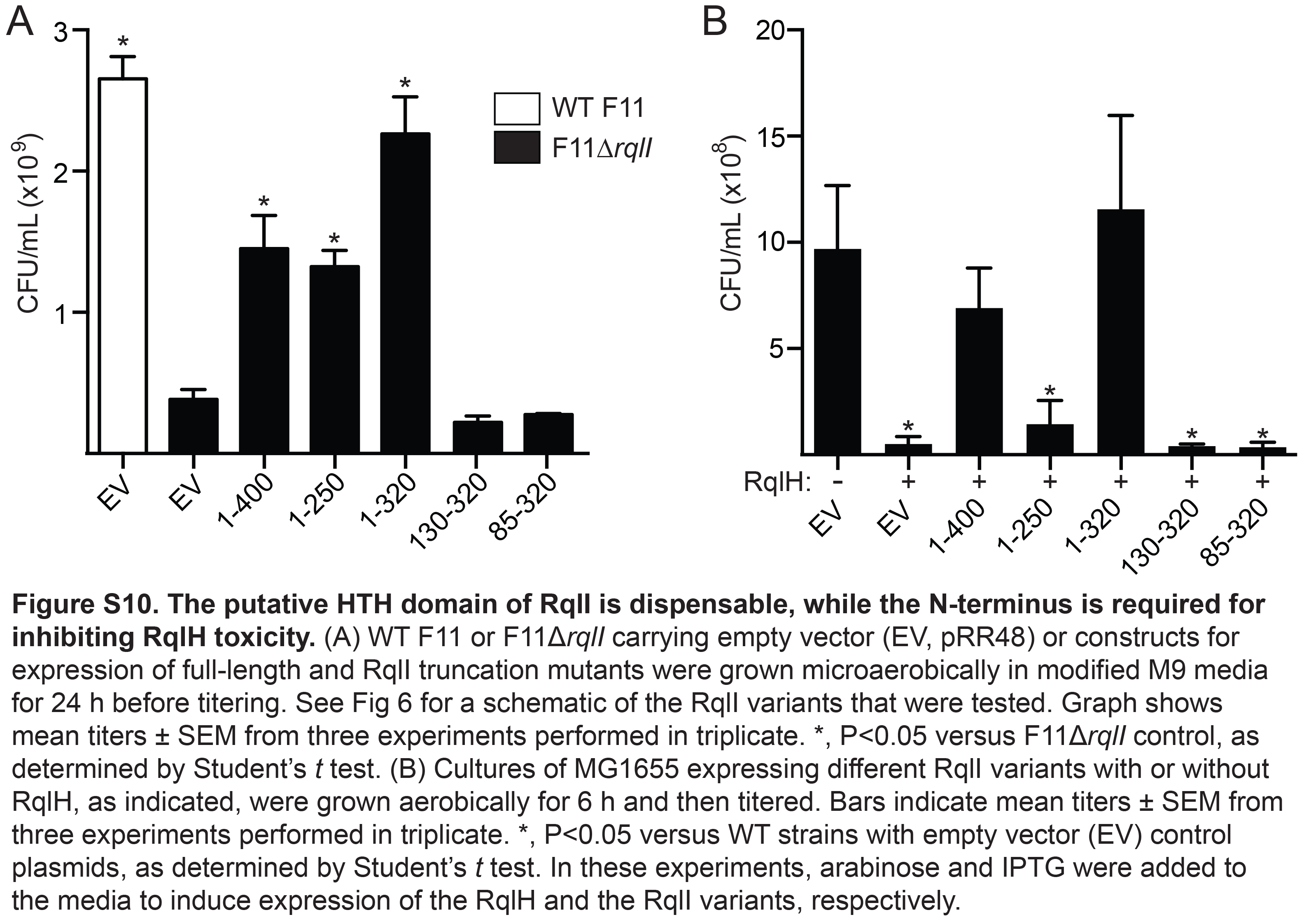

Supplement: S10 Fig — (A) WT F11 or F11ΔrqlI carrying empty vector (EV, pRR48) or constructs for expression of full-length and RqlI truncation mutants were grown microaerobically in modified M9 media for 24 h before titering. See Fig 6 for a schematic of the RqlI variants that were tested. Graph shows mean titers ± SEM from three experiments performed in triplicate. *, P<0.05 versus F11ΔrqlI control, as determined by Student’s t test. (B) Cultures of MG1655 expressing different RqlI variants with or without RqlH, as indicated, were grown aerobically for 6 h and then titered. Bars indicate mean titers ± SEM from three experiments performed in triplicate. *, P<0.05 versus MG1655 with empty vector (EV) control plasmids, as determined by Student’s t test. In these experiments, arabinose and IPTG were added to the media to induce expression of the RqlH and the RqlI variants, respectively. (TIF) [file ppat.1005317.s014.tif]

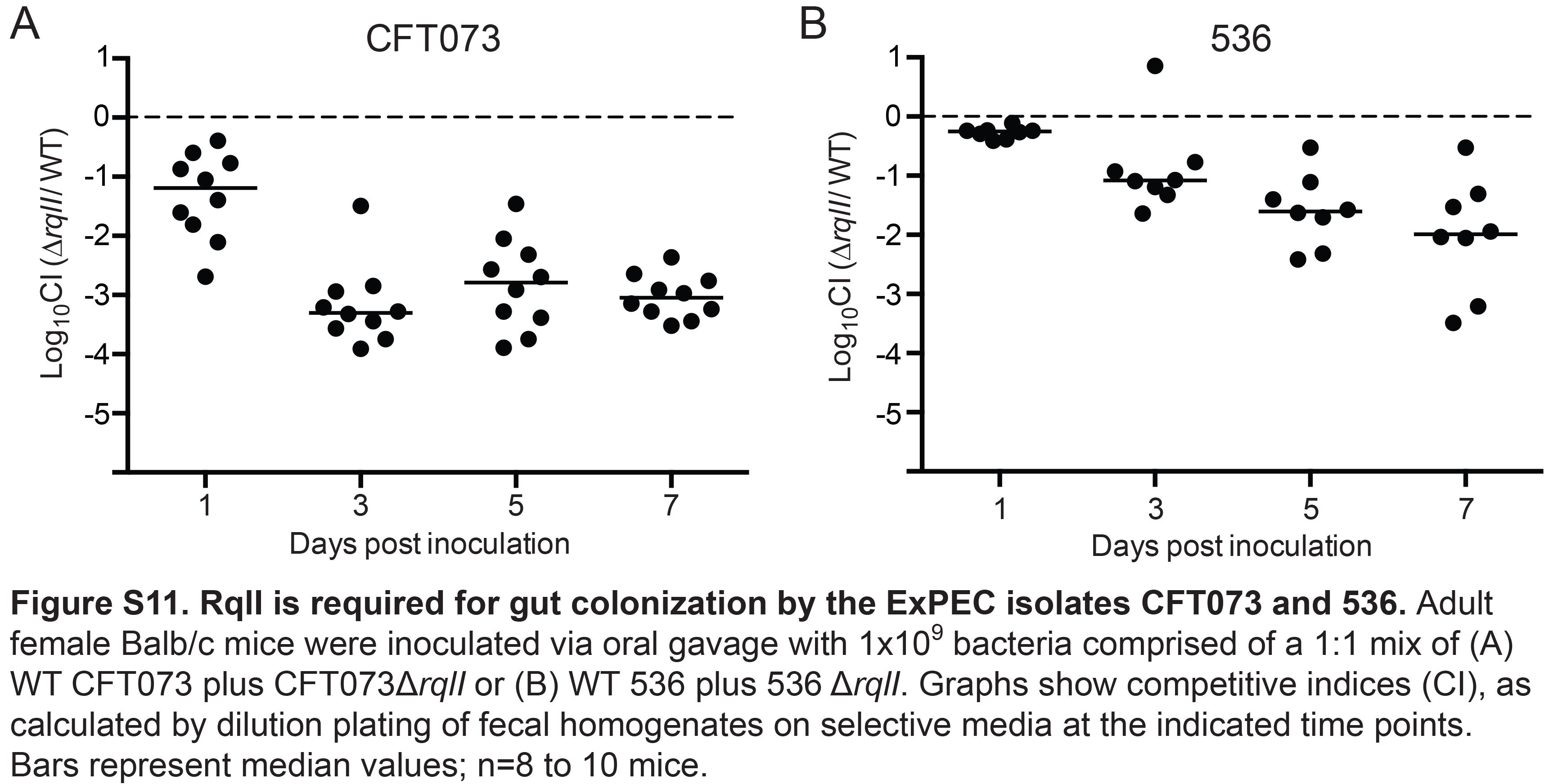

Supplement: S11 Fig — Adult female Balb/c mice were inoculated via oral gavage with 1x10^9^ bacteria comprised of a 1:1 mix of (A) WT CFT073 plus CFT073ΔrqlI or (B) WT 536 plus 536 ΔrqlI. Graphs show competitive indices (CI), as calculated by dilution plating of fecal homogenates on selective media at the indicated time points. Bars represent median values; n = 8 to 10 mice. (TIF) [file ppat.1005317.s015.tif]

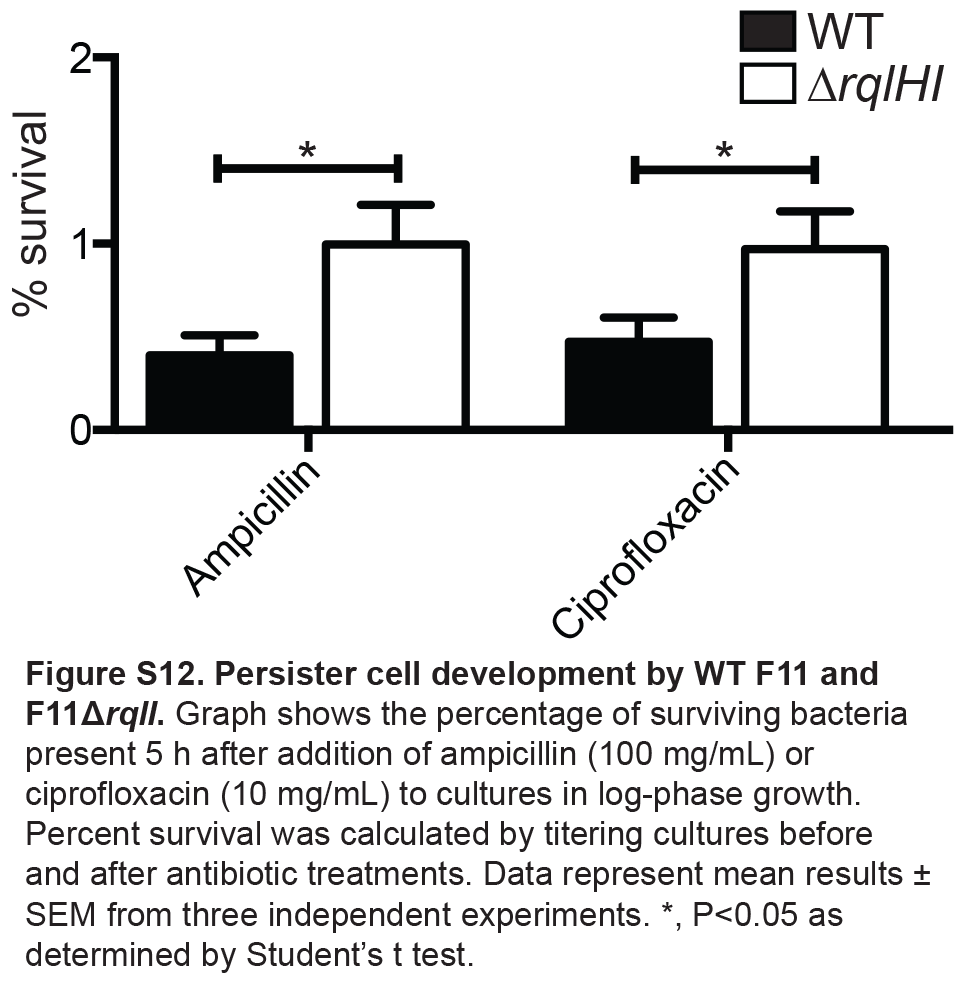

Supplement: S12 Fig — Graph shows the percentage of surviving bacteria present 5 h after addition of ampicillin (100 mg/mL) or ciprofloxacin (10 mg/mL) to cultures in log-phase growth. Percent survival was calculated by titering cultures before and after antibiotic treatments. Data represent mean results ± SD from three independent experiments. *, P<0.05 as determined by Student’s t test. (TIF) [file ppat.1005317.s016.tif]
